# Supplementary material for: Analysis of Genetic Relatedness between Gastric and Oral Helicobacter pylori in Patients with Early Gastric Cancer Using Multilocus Sequence Typing
Source: Int J Mol Sci. 2023 Jan 22;24(3):2211. doi: 10.3390/ijms24032211 (PMC9917182; doi:10.3390/ijms24032211)
Supplement: Supplementary file 1 [file ijms-24-02211-s001.zip › Supplementary_Figure_S2.pdf]

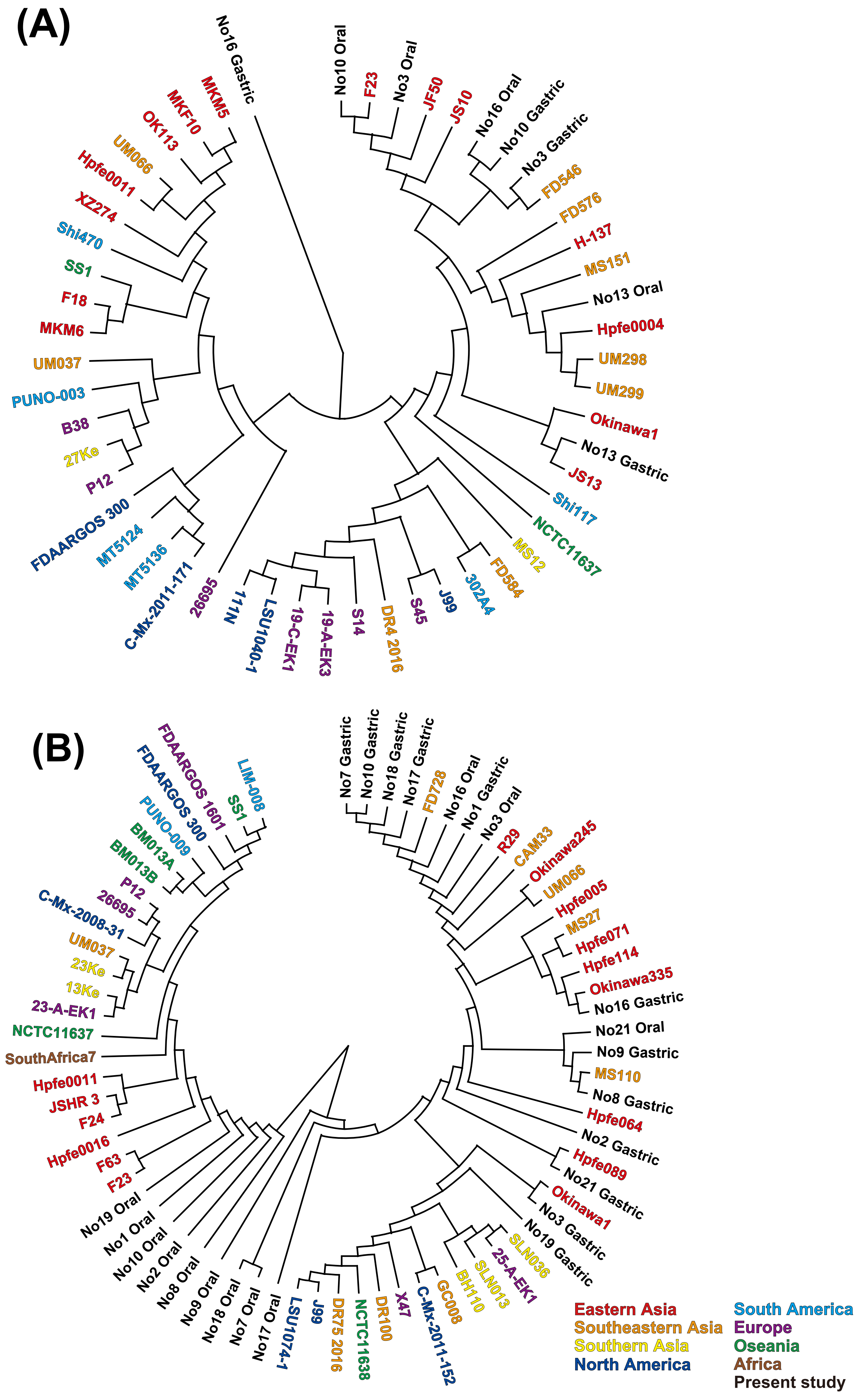
(A)
No16 Gastric
No10 Oral
F23
No3 Oral
JF50
JS10
No16 Oral
No10 Gastric
No3 Gastric
FD546
FD576
H-137
MS151
No13 Oral
Hpfe0004
UM298
UM299
Okinawa1
No13 Gastric
JS13
Shi117
NCTC11637
MS12
FD584
302A4
J99
S45
DR4 2016
S14
19-A-EK3
19-C-EK1
LSU1040-1
111N
26695
C-Mx-2011-171
MT5136
MT5124
FDAARGOS 300
P12
27Ke
B38
PUNO-003
UM037
MKM6
F18
SS1
Shi470
XZ274
Hpfe0011
UM066
OK113
MKF10
MKM5
(B)
No7 Gastric
No10 Gastric
No18 Gastric
No17 Gastric
FD728
No16 Oral
No1 Gastric
No3 Oral
R29
CAM33
Okinawa245
UM066
Hpfe005
MS27
Hpfe071
Hpfe114
Okinawa335
No16 Gastric
No21 Oral
No9 Gastric
MS110
No8 Gastric
Hpfe064
No2 Gastric
Hpfe089
No21 Gastric
Okinawa1
No3 Gastric
No19 Gastric
25-A-EK1
SLN036
SLN013
BH110
GC008
C-Mx-2011-152
X47
DR100
NCTC11638
DR75 2016
J99
LSU1074-1
No17 Oral
No7 Oral
No18 Oral
No9 Oral
No8 Oral
No2 Oral
No10 Oral
No1 Oral
No19 Oral
F23
F63
Hpfe0016
F24
JSHR 3
Hpfe0011
SouthAfrica7
NCTC11637
23-A-EK1
13Ke
23Ke
UM037
C-Mx-2008-31
26695
P12
BM013B
BM013A
PUNO-009
FDAARGOS 300
FDAARGOS 1601
SS1
LIM-008
Eastern Asia
Southeastern Asia
Southern Asia
North America
South America
Europe
Oseania
Africa
Present study

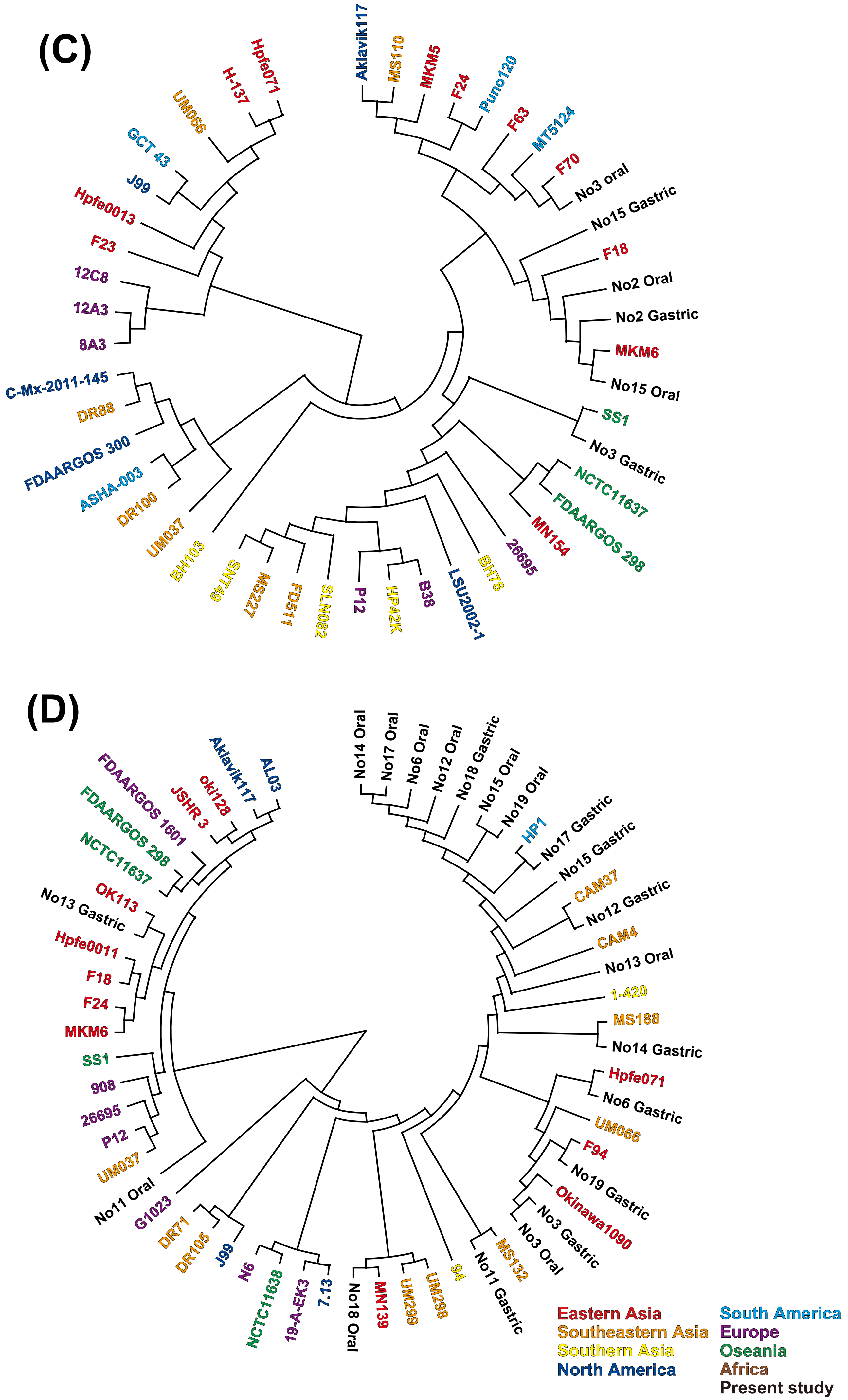
(C)
Aklavik117
MS110
MKM5
F24
Puno120
F63
MT5124
F70
No3 oral
No15 Gastric
F18
No2 Oral
No2 Gastric
MKM6
No15 Oral
SS1
No3 Gastric
NCTC11637
FDAARGOS 298
MN154
26695
BH78
LSU2002-1
B38
HP42K
P12
SLN082
FD511
MS227
SNT49
BH103
UM037
DR100
ASHA-003
FDAARGOS 300
DR88
C-Mx-2011-145
8A3
12A3
12C8
F23
Hpfe0013
J99
GCT 43
UM066
H-137
Hpfe071
(D)
No14 Oral
No17 Oral
No6 Oral
No12 Oral
No18 Gastric
No15 Oral
No19 Oral
HP1
No17 Gastric
No15 Gastric
CAM37
No12 Gastric
CAM4
No13 Oral
1-420
MS188
No14 Gastric
Hpfe071
No6 Gastric
UM066
F94
No19 Gastric
Okinawa1090
No3 Gastric
No3 Oral
MS132
No11 Gastric
94
UM298
UM299
MN139
No18 Oral
7.13
19-A-EK3
NCTC11638
N6
J99
DR105
DR71
G1023
No11 Oral
UM037
P12
26695
908
SS1
MKM6
F24
F18
Hpfe0011
No13 Gastric
OK113
NCTC11637
FDAARGOS 298
FDAARGOS 1601
JSHR 3
oki128
Aklavik117
AL03
Eastern Asia
South America
Southeastern Asia
Europe
Southern Asia
Oseania
North America
Africa
Present study

(E)

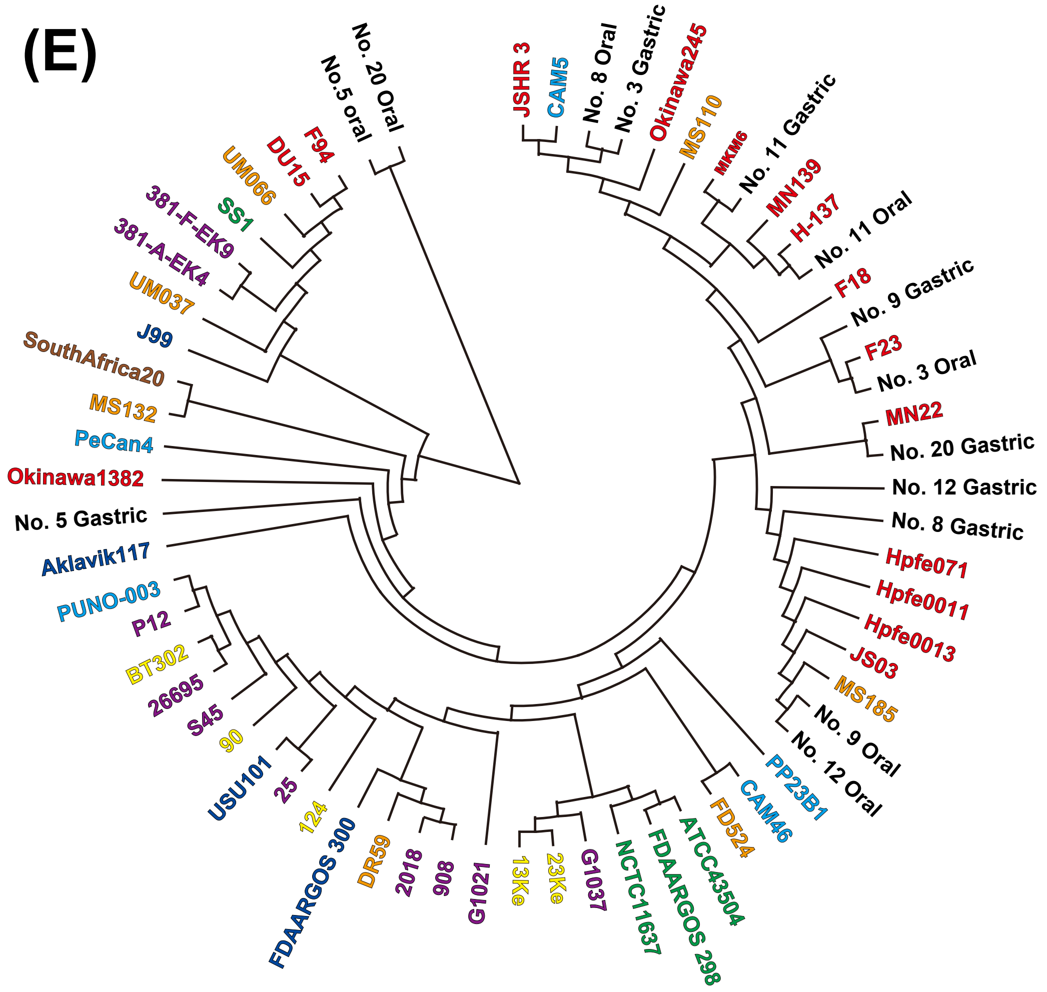
No. 20 Oral
No.5 oral
F94
DU15
UM066
SS1
381-F-EK9
381-A-EK4
UM037
J99
SouthAfrica20
MS132
PeCan4
Okinawa1382
No. 5 Gastric
Aklavik117
PUNO-003
P12
BT302
26695
S45
90
USU101
25
124
FDAARGOS 300
DR59
2018
908
G1021
13Ke
23Ke
G1037
NCTC11637
FDAARGOS 298
ATCC43504
FD524
CAM46
PP23B1
No. 12 Oral
No. 9 Oral
MS185
JS03
Hpfe0013
Hpfe0011
Hpfe071
No. 8 Gastric
No. 12 Gastric
No. 20 Gastric
MN22
No. 3 Oral
F23
No. 9 Gastric
F18
No. 11 Oral
H-137
MN139
No. 11 Gastric
MKM6
MS110
Okinawa245
No. 3 Gastric
No. 8 Oral
CAM5
JSHR 3

(F)

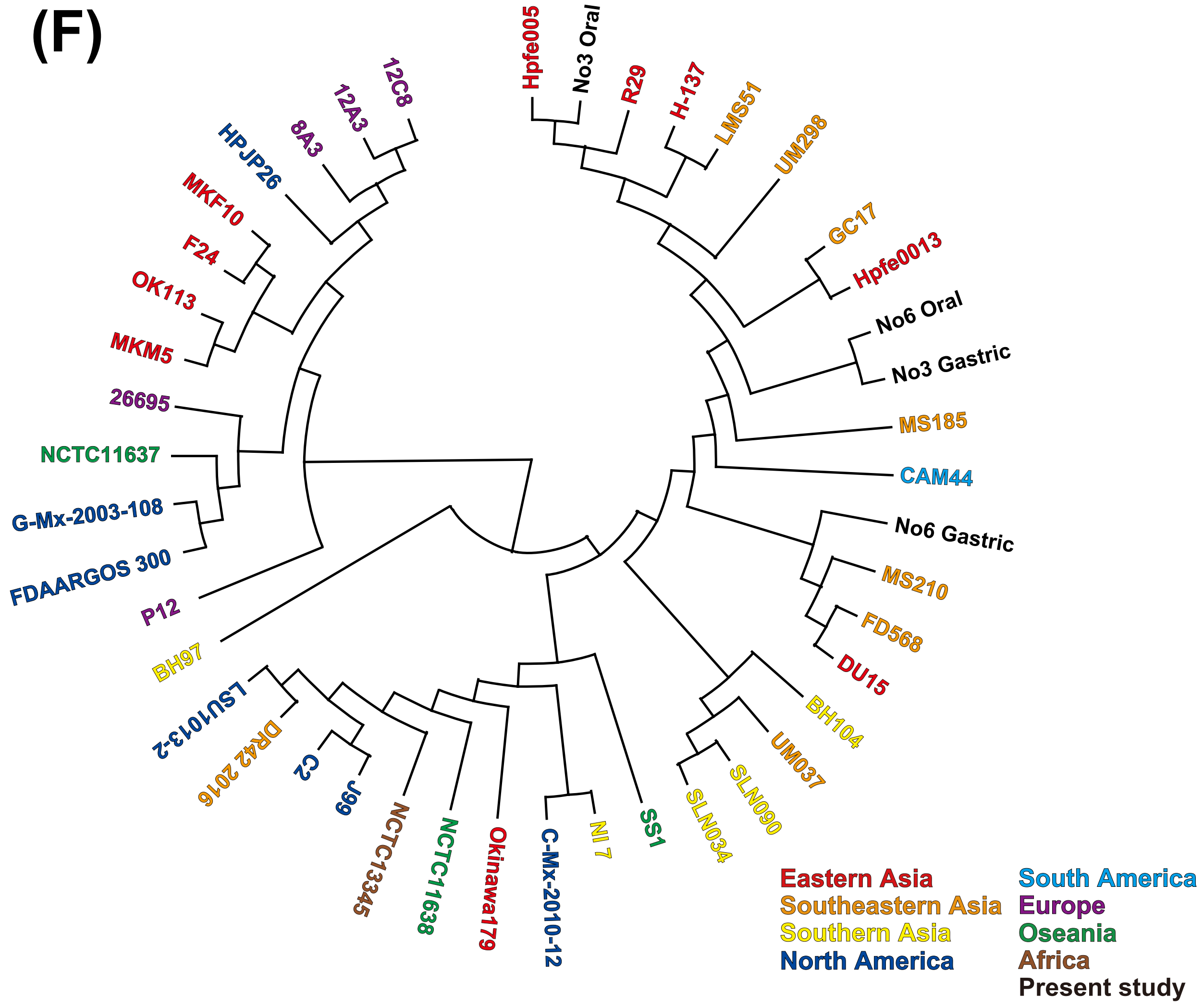
Hpfe005
No3 Oral
R29
H-137
LMS51
UM298
GC17
Hpfe0013
No6 Oral
No3 Gastric
MS185
CAM44
No6 Gastric
MS210
FD568
DU15
BH104
UM037
SLN090
SLN034
SS1
NI 7
C-Mx-2010-12
Okinawa179
NCTC11638
NCTC13345
J99
C2
DR42 2016
LSU1013-2
BH97
P12
FDAARGOS 300
G-Mx-2003-108
NCTC11637
26695
MKM5
OK113
F24
MKF10
HPJP26
8A3
12A3
12C8
Eastern Asia
South America
Southeastern Asia
Europe
Southern Asia
Oceania
North America
Africa
Present study

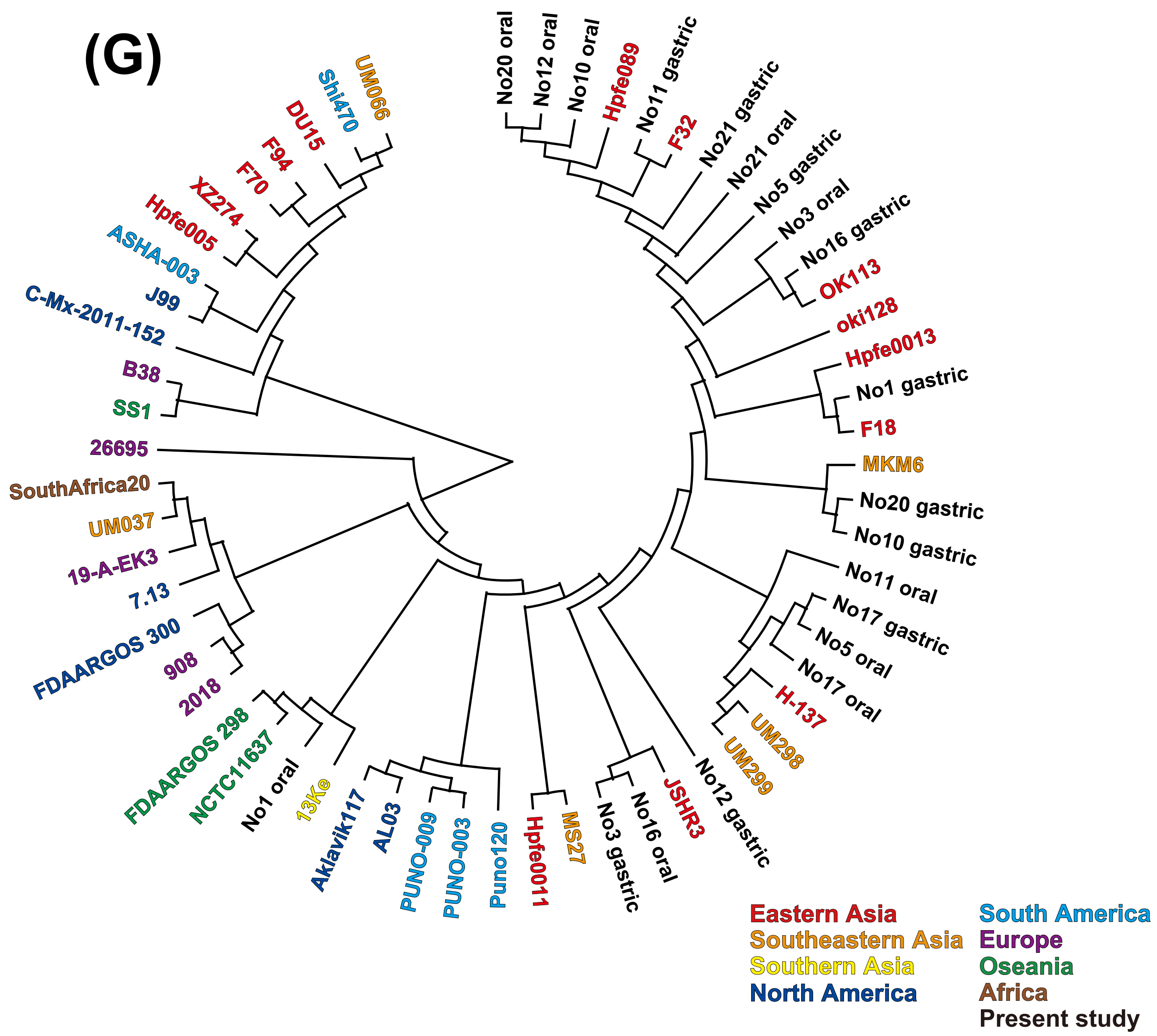
(G)
No20 oral
No12 oral
No10 oral
Hpfe089
No11 gastric
F32
No21 gastric
No21 oral
No5 gastric
No3 oral
No16 gastric
OK113
oki128
Hpfe0013
No1 gastric
F18
MKM6
No20 gastric
No10 gastric
No11 oral
No17 gastric
No5 oral
No17 oral
H-137
UM298
UM299
No12 gastric
JSHR3
No16 oral
No3 gastric
MS27
Hpfe0011
Puno120
PUNO-003
PUNO-009
AL03
Aklavik117
13Ke
No1 oral
NCTC11637
FDAARGOS 298
2018
908
FDAARGOS 300
7.13
19-A-EK3
UM037
SouthAfrica20
26695
SS1
B38
C-Mx-2011-152
J99
ASHA-003
Hpfe005
XZ274
F70
F94
DU15
Shi470
UM066
Eastern Asia
Southeastern Asia
Southern Asia
North America
South America
Europe
Oseania
Africa
Present study
