# Supplementary material for: Analysis of Genetic Relatedness between Gastric and Oral Helicobacter pylori in Patients with Early Gastric Cancer Using Multilocus Sequence Typing
Source: Int J Mol Sci. 2023 Jan 22;24(3):2211. doi: 10.3390/ijms24032211 (PMC9917182; doi:10.3390/ijms24032211)
Supplement: Supplementary file 1 [file ijms-24-02211-s001.zip › Supplementary_file_S1.pdf]

>NCTC11637

AATGAGTTCAGTCTCATCGAGCAAAAAGCCCCAGGCATTATGGACAGAAAATCTGTGCAT
GAGCCTTTGCAAACTGGCATTAAAGCCATTGATGCGTTGGTGCCTATTGGACGCGGGCAA
AGGGAATTGATTATTGGTGATAAACAAACCGGTAAAACCACCGTAGCGATCGATGCGATC
ATTAACCAAAAAGGGCAAAATGTGATCTGTATCTATGTGGCTATTGGGCAAAAAGAATCC
ACTGTCGCGCAAGTGGTCCGCAAATTAGAAGAATACGGGGCGATGGAATACAGCGTCGT
GATCAACGCTTCGGCTTCAGATTCAGCTGCGATGCAATATTTAGCCCCTTATTCAGGTGTG
GCTATGGGGGAATACTTTAGAGATCATGCCCGCCATGCCCTAATCATTTATGATGATTTGAG
TAAGCATGCTGTCGCTTACAGAGAAATTTCTTTGATTTTGAGAAGACCCCCAGGTAGGGA
GGCTTTTCCTGGAGATGTGTTTTATATCCACTCACGGCTTTTAGAAAGAGCGGCTAAACTT
TGCGATGAAAAGGGTGCCGGCTCTTTGACCGCGCTCCCTATTGTGGAAACTCAAGCGGG
CGATGTTTCAGCTTATATCCCTACGTACCAGCATGTCAAGCCCGGCAAGGGTGCGGCTTTT
GTGCGTGCGAAAATCAAGTCGTTTTTAGATGGCAAGGTGATTGAGAAGACTTTCCATGCG
GGGGATAAGTGCGAAGAGCCTAATCTGGTTGAAAAAACGATGCAATACCTTTATCACGAT
GGCGACACATACCAATTCATGGATATAGAGAGCTATGAGCAAATCGCTTTGAACGACTCT
CAAGTGGGTGAGGCTTCTAAATGGATGCTAGACGGCATGCAAGTGCAGGTTTTATTGCAT
AATGACAAGGCGATTTCAGTGGATGTGCCGCAAGTTGTGGCTCTAAAGATTGTAGAAAC
AGCCCCTAATTTTAAGGGCGATACTTCAAGTGCAAGCAAAAAACCAGCGACTTTAGAAA
CCGGTGCGGTCGTGCA

AGGGGAGCAGATTAAAGCCCCTAGATCAATTAGGGCTTGGTTATGATTAAAGCTTTCATTA
AGATTGAGAAAGCCATTCGCTTTAATTTGTAAGTCTTTAGCCGTGATATTAGGATCCAAAC
CAAAAAGCCTTAAAAGCACTCGTTTGATATTAGCATCCACGCATGCGCTCTTTTCTCTAAA
GCCAAAACATAAAATGGCATTAGCCGTGTATGCGCCAATTCCTGGGAGTTTCAATAGGCT
TTGATAGTCATTAGGTAATTGTGAGTTATGTTCTTTAACGCAAATTTCAGCGCTTTTTTTA
AATTTTTAGCCCTTGAATAATAACCAAGCCCTCTCCAGAGCAATAAAACCTCCTCTAATGG
AGCGTTCGCTAAGTCTTTTAAAGTGGGGAAAGCTTCTAAAAAGGGGGAATAAAA

ATTCTAATATCAAGTATGAATTGGATAAAGAAAGCGGGGCTTTAATGGTGGATAGGGTGCT
TTATGGGGCGCAAAATTACCCTGCGAATTACGGCTTTGTGCCTAACACTTTAGGATCTGAT
GGCGACCCTGTAGATGCGCTGGTTTTAAGCGATGTGGCTTTTCAAGCCGGGAGCGTGGT
GAAAGCGCGCTTGGTTGGGGTTTTGAACATGGAAGATGAAAGCGGAGTGGATGAAAAAT
TACTCGCTCTACCCATAGATAAGATCGATCCCACGCATTCCTATGTCAAAGATATTGATGAT
TTATCCAAACACACTTTAGATAAAATCAAACATTTTTTTGAAACTTACAAGGATTTAGAGC
CTAATAAATGGGTGAAAGTCAAGGGGTTTGAAAACTAAAAAGCCATTCACATAAGGGGC
TAGGGCTTTGATTTGTACATGCGAATGAATGCCGGACTCGCTCACCACGAGCGCGTCTTT
AGGCAAGAGAGAGTGCAATTTGAGCGTGTTGTTAATGTCGGTTGTCAGGGTGTGCAAAT
CCCTGTTATTGATGCCTATAATGTCGTATTGGAGTTTGAGCAAGCGCTCAATTTCTTGCTG

GTTGGAAACTTCAGTTAACACGCTCATGTTTAAGGATTTGGCGAGGTTGAAAAGCTCTAA
ATAATTTTTATCATCTAACGCACTTAACATTAAAAGCACCGCATTAGCTCCCATCATTCTAG
CGAGTTTGATCTGAAAAGCGTCAATGATAAAATCTTTACACAAAATAGGCTTGGTGGAAT
GCTGCGAAACGATCTTAATGTTTTCATAAGAGCCTAAAAAATATTTAGAATCAGCTAAAAC
TGAAATGCACACCCAGTGTTGGATAAAGAGTAACCAAGCAGGGATCCAAGCGGTTAAAA
TACCCTCAATGATAGTAAGCCATGGAGTGAATTTCCCTAAAGGGATTTTCAAGATGTTTTC
AATGAAAGCGGTAAGCCACAAAACACCCCAAGCCAACCAAATGATCGCCCACCAATCGC
CTTCAGTGATGCCTAACACTTTGTGGTCATCAAGCATATCGCTATAGTGGGATAAAATCGC
AGCAGGAATCGCGTTGATCGCTACGAATAAGCTATACCAAGAATAAGGCCTCCAATCCAA
ACCAAAAGTGTGGTTGATAGCCGCATACAAGTAGGTAAAACCGAACAATAACCCAGTCG
CTGGCCCATAGAAATTAGTCAAATGGTGCGATACTTGAGCGATATCTTCAGCACCTTCTAC
AGGGGCTGTAGGGTTGAGAGCGGAATAAGTGATGACAACCACATTACAAACAATGGAAA
GCCCGCCCACAAAAAAGTTCATCACCGCAGTGCTTTTAGGATCGACTTTGGTTAACCCGC
AAATCCCATTGCTGATTAAAACAATCCCAAGAAGAAGCCAATAAAACCCCAGATAAACCC
GATAAAGTTTGGCGCATTCAAGCAGGAAGAGGCTTCAATAATTTTCCTCACAAGGAATAC
GACTTATACAAATCCCTTTTATCCAGTAAGATTGATGGAGGCTGGGATTGGGGGAATGCC
GCTAGGCATTATTGGGTCAAAGGCGGGCAATGGAACAAGCTTGAAGTGGATATGAAAGA
CGCTGTAGGGACTTATAAACTTTCAGGCCTTATCAACTTTACTGGTGGGGATTTAGATGTC
AATATGCAAAAAGCCACTTTGCGCTTGGGCCAATTCAATGGCAATTCTTTCACAAGCTATA
AGGATAGTGCTGATCGCACCACGAGAGTGGATTTCAACGCTAAAAATATCTTAATTGATA
ATTTTTTAGAAATCAATAATCGTGTGGGTTCTGGAGCCGGGAGGAAAGCCAGCAATACAA
GCCCTAAAACTTTAAAAACCATTGCGATTTTAGGCCAGCCTAATGTGGGGAAAAGCTCGT
TATTTAACCGCTTAGCTAGAGAAAGGATCGCTATCACTTCAGATTTTGCAGGCACTACACG
AGACATTAACAAACGAAAAATCGCATTGAATGGCCATGAAGTGGAATTGCTAGATACAGG
GGGCATGGCTAAAGGCGCTCTTTTGTCTAAAGAAATCAAAGCCATTAATTTAAAAGCCGC
TCAAATGAGCGATTTGATTTTATACGTTGTGGATGGCAAGTCTATCCCTAGCGATGAAGAC
ATCAAGCTTTTTAGAGAGGTTTTTAAGATCAACCCTAACTGCTTTTTAGTGATCAATAAGA
TTGATAACGACAAAGAAAAAGAGCGAGCTTATGCGTTTTCTTCTTTTGGCATTCCCAAGA
GTTTTAACATCTCCGTTTCGCACAATAGAGGCATTAACGCATTAATTGATGCAATATTGAG
GGCGCTGGATTTAAACCAAATCATAGAGCAGGATTTGGAT
>oki128
CGTAGGGATATAAGCTGAAACATCGCCCGCTTGAGTTTCCACAATAGGGAGTGCGGTCAA
AGAGCCTGCACCCTTTTCATCGCAAAGTTTAGCCGCTCTTTCTAAAAGCCGTGAGTGGAT
ATAAAACACATCTCCAGGAAAAGCCTCCCTACCTGGAGGTCTTCTCAAAATCAAAGAAA
TTTCTCTGTAAGCGACAGCATGCTTACTCAAATCATCATAAATGATTAGGGCATGGCGGGC
ATGATCTCTAAAGTATTCCCCCATAGCCACACCTGAATAAGGGGCTAAATATTGCATCGCA

GCTGAATCTGAAGCCGAAGCGTTGATCACGACGCTGTATTCCATCGCTTCGTATTCTTCTA
ATTTGCGGACCACTTGCGCGACAGTGGATTCTTTTTGCCCAATAGCCACATAGATACAGAT
CACATTTTGCCCTTTTTGGTTAATGATCGTATCGATCGCTACGGTGGTTTTACCGGTTTGTT
TGTCCCCAATGATCAATTCCCTTTGCCCACGCCCAATAGGCACCAACGCATCAATGGCTTT
AATGCCTGTTTGCAAAGGTTCATGCACCGATTTTCTGTCCATAATGCCCGGGGCTTTTTGC
TCAATGAGGCTAAATTCATTTACCAACATGTCAAGCCCGGCAAGGGCGCAGCTTTTGTGC
GCGCGAAAATCAAGTCGTTTTTAGATGGCAAGGTGATTGAAAAGACTTTCCATGCGGGG
GATAAGTGCGAAGAGCCTAATCTGGTTGAAAAAACGATGCAATACCTTTATCACGATGGC
GATACATACCAATTCATGGACATAGAGAGCTATGAGCAAATCGCCTTAAACGACTCTCAA
GTGGGCGAGGCTTCTAAATGGATGCTAGACGGCATGCAAGTGCAGGTTTTATTGCATAAT
GACAAGGCGATTTCAGTGGATGTGCCGCAAGTTGTGGCTCTAAAGATTGTAGAAACAGC
CCCTAATTTTAAGGGCGATACTTCGAGCGCGAGCAAAAAACCAGCGACTTTAGAAACCG
GTGCGGTCGTGCAAGGGGAGCAGATTAAAGCCCCTAGATCAATTAGGGCTTGGTTATGAT
TAAAACTTTCATTAAGATTGAGAAAGTCATTCGCCTTAATTTGTAGATCTTTAGCCGTGAT
ATTAGGATCCAAACCAAAAAGCCTTAAAAGCGTTCGTTTGATGTTAGCATCCACGCATGC
GCTCTTTTCTCTAAAGCCAAAACATAAAATCGCATTAGCCGTGTATGCGCCAATCCCGGG
GAGTTTTAACAGGCTTTGATAGTCATTGGGTAATTGTGAGTTATGTTCTTTAGCGCAAATT
TCAGCGCTTTTTTTTAAATTTTTAGCCCTTGAATAATAGCCAAGCCCTCTCCAGAGCAATA
AAACCTCTTCTAATTGAGCGTTCGCTAAGTCTTTTAAAGTGGGGAAAGCTTCTAAAAAAG
GGGAATAAAA
ATTCTAATATTAAATATGAATTGGATAAAGAAAGTGGGGCTTTAATGGTGGATAGGGTGCT
TTATGTGGCGCAAAATTACCCCGCCAATTATGGCTTTGTGCCTAACACTCTAGGATCTGAT
GGCGACCCTGTAGATGCGCTTGTTTTAAGCGATGTGGCTTTTCAAGCCGGAAGCGTGGTG
AAAGCGCGCTTGGTTGGGGTTTTGAACATGGAAGATGAAAGCGGAATGGATGAAAAATT
ACTCGCTCTGCCCATAGATAAGATCGATCCCACGCATTCCTATGTCAAAGATATTGATGATT
TATCCAAACACACTTTAGATAAAATCAAGCATTTTTTTGAAACTTACAAGGATTTAGAGCC
TAATAAATGGGTGAAAGTCAAGGGGTTTGAAAACTGCATTTCAGTTTTAGCCGATTCTAA
ATATTTTTTAGGCTCTTATGAAAACATTAAGATGGTTTCGCAACATTCCATTAAGCCCATTT
TATGCAAAGATTTTATCATTGATTCTTTTCAGATCAAACTCGCTAGAATGATGGGGGCTAA
TGCGGTGCTTTTAATGTTAAGCGTATTGGATGATAAAAATTATTTAGAGCTTTTCAATCTCG
CTAAATCCTTAAACATGAGCGTTCTGACTGAAGTTTCTAACCAGCAAGAAATCAAGCGCT
TGCTCAAACTCCAATACGACATTATCGGCATCAATAACAGGGATTTACACACCTTAAAAA
CCGATATTGATCACACGCTCAAATTACGCCCCCTTTTGCCTAAAGACGCGCTCATTATCAG
CGAGTCCGGTATTTATTCGCACACGCAAATCAAAGCCCTAGCCCCTTATGTGAATGGCTTT
TTACACCCAGTGTTGGATAAAGAGTAACCAAGCGGGGATCCAAGCGGTTAAAATACCCTC
AATGATAGCAAGCCATGGAGTGAATTTCCCTAAAGGAATTTTCAAGATGTTTTCAATGAA

AGCGGTAAGCCATAAAACACCCCAAGCCAACCAAATGATCGCCCACCAATCGCCTTCAG
TGATGCCTAACACTTTGTGGTCATCAAGCATATCGCTATAGTGGGATAAAATCGCAGCAGG
AACAGTGTTGATCGCTACGAATAAGCTATACCAAGAGTAGGGCCTCCAATCCAAACCAAA
AGTGTGGTTGATAGCCGCATACAAGTAGGTAAAACCGAACAATAACCCAGTTGCTGGCCC
ATAGAAACTAGTCAAATGGTGCGATACTTGAGCAATATCTTCTGCGCCTTCTACAGGGGCT
GTAGGGTGGAGAGCAGAATAAGTGATGACAACCACATTACAAACAATGGAAAGCCCACC
CACAAAAAAGTTCATCACCGCAGTGCTTTTAGGATCGACTTTGGTTAATCCGCAAATCCC
GTTGCTGATTAAAACAATCCCAAGAACAAGCCAATAAAACCCCGGATAAACCCGATAAA
GTTTGGCGCATTCAAGCAGGAAGAGGCTTTAATAATTTCCCTAACAAGCAATACGACTTA
TACAAATCCCTTTTATCCAGTAAGATTGATGGAGGCTGGGATTGGGGGAATGCCGCTAGG
CATTATTGGGTTAAAGATGGGCAATGGAATAAGCTTGAAGTGGATATGCAAGACGCTGTA
GGGACTTATAACCTTTCAGGGCTTATCAACTTTACCGGTGGGGATTTAGATGTCAATATGC
AAAAAGCCACTTTGCGTTTGGGCCAATTCAATGGCAATTCTTTCACAAGCTTTAAGGATG
CGGCTGATCGCACCACGAGAGTGAATTTTGACGCTAAAAATATCTTAATTGATAATTTTTT
AGAAATCAATAATCGTGTGGGTTCTGGAGCCGGCAGGAAAGCCAGC
TTTGATTAAAGTGAAACTACAAGTAAAATGAATACAAGCCATAAAACTTTAAAAACCATT
GCGATTTTAGGCCAGCCTAATGTGGGGAAAAGCTCGTTATTTAACCGCTTGGCTAGAGAA
AGGATCGCTATCACTTCAGATTTTGCAGGCACTACACGAGACATTAACAAACGAAAAATC
GCATTGAATGGCCATGAAGTGGAATTGCTAGATACAGGGGGCATGGCTAAAGACGCTCTT
TTGTCTAAAGAAATCAAAGCCCTTAATTTAAAAGCCGCTCAAATGAGCGATTTGATTTTAT
ATGTTGTGGATGGCAAGTCTATCCCTAGCGATGAAGATCTTAAGCTTTTCAGAGAGGTTTT
TAAAACCAACCCTAACTGCTTTTTAGTGATCAATAAAATTGATAACGACAAAGAAAAAGA
GCGATCTTATGCGTTTTCTTCTTTTGGCATGCCAAAGAGTTTTAACATTTCCGTTTCGCAC
AATAGAGGCATTAGCGCCTTAATTGATGCGGTATTGAATGCGCTAAATTTAAATCAAATCA
TAGAGCAGGATTTGGATGCAGATATTTTAGAAAGCCTAGAAAATAACGCATCAAAAGA
>F32
CGTAGGGATATAGGCTGAAACATCGCCCGCTTGAGTTTCCACAATAGGGAGCGCGGTCAA
AGAACCCGCACCCTTTTCATCGCAAAGTTTAGCCGCTCTTTCTAAAAGCCGTGAGTGGAT
ATAAAACACATCTCCAGGAAAAGCCTCCCTACCTGGAGGTCTTCTCAAAATCAAAGAAA
TTTCTCTGTAAGCGACAGCATGCTTACTCAAATCATCATAAATGATTAGGGCATGGCGGGC
ATGATCTCTAAAGTATTCCCCCATAGCCACACCTGAATAAGGGGCTAAATATTGCATCGCA
GCTGAATCTGAAGCCGAAGCGTTGATCACGACGCTGTATTCCATCGCTCCGTATTCTTCTA
ATTTGCGGACCACTTGCGCGACAGTGGATTCTTTTTGCCCAATAGCCACATAGATACAGAT
CACATTTTGCCCTTTTTGGTTAATGATCGCATCGATCGCTACGGTGGTTTACCGGTTTGTT
TGTCCCCAATGATCAATTCCCTTTGCCCGCGCCCAATAGGCACCAACGCATCAATGGCTTT
AATGCCTGTTTGCAAAGGCTCATGCACCGATTTTCTGTCCATGATGCCCGGGGCTTTTTGC

TCGATGAGGCTAAACTCATTTACCAGCATGTCAAGCCCGGCAAGGGTGCGGCTTTTGTGC
GCGCGAAAATCAAGTCGTTTTTAGATGGTAAGGTGATTGAAAAGACTTTCCATGCGGGG
GATAAGTGCGAAGAGCCTAATTTGGTTGAAAAAACGATGCAATACCTTTATCATGATGGC
GATACATACCAATTCATGGACATAGAGAGCTATGAGCAAATCGCTTTGAACGACTCTCAA
GTGGGTGAGGCTTCTAAATGGATGCTAGACGGCATGCAAGTGCAGGTTTTATTGCATAAT
GACAAGGCGATTTCAGTGGATGTGCCGCAAGTTGTGGCTTTAAAAATTGTAGAAACAGC
CCCTAATTTTAAGGGCGATACTTCAAGTGCGAGCAAAAAGCCAGCGACTTTAGAAACCG
GTGCGGTCGTGCA

GGGGAGCAGATTAAAGCCCCTAGATCAATTAGGGCTTGGTTATGATTAAAGCTTTCATTAA
GATTGAGAAAGTCATTCGCCTTAATTTGTAGATCTTTAGCCGTGATATTTGGATCCAAACC
AAAAAGCCTTAAAAGCACTCGTTTGATATTGGCATCCACGCATGCACTCTTTTCTCTAAA
GCCAAAACATAAAATCGCATTAGCCGTGTATGCGCCAATCCCGGGGAGTTTTAACAGGCT
TTGATAGTCATTGGGTAATTGTGAGTTGTGTTCTTTAACGCAAATTTCAGCGCTTTTTTTA
AATTTTTAGCCCTTGAATAATAGCCAAGCCCTCTCCAAAGCAATAAAACCTCCTCTAATTG
AGCGTTCGCTAAGTCTTTTAAAGTGGGGAAAGCTTTTAAAAAAGGGGAATAAAA
ATTCTAATATCAAGTATGAACTGGATAAAGAAAGCGGGGCTTTAATGGTGGATAGGGTGC
TTTATGGGGCGCAAAATTACCCCGCCAATTATGGCTTTGTGCCTAACACTTTAGGATCTGA
TGGCGACCCTGTAGATGCGCTTGTTTTAAGCGATGTGGCTTTTCAAGCCGGGAGCGTAGT
GAAAGCGCGTTTGGTTGGGGTTTTGAACATGGAAGATGAAAGCGGAATGGATGAAAAAT
TACTCGCTCTACCCATAGATAAGATCGATCCCACGCATTCCTATGTCAAAGACATTGATGA
TTTATCCAAACATACTTTAGATAAAATCAAGCATTTTTTTGAAACTTACAAGGATTTAGAG
CCTAATAAATGGGTGAAAGTCAAGGGGTTTGAAAACTAAAAAGCCATTCACATAAGGGG
CTAGGGCTTTGATTTGCGCGTGCGAATAAATACCGGACTCGCTGATAATGAGCGCGTCTTT
AGGTAAAAGGGGGCGCAATTTGAGCGTGTGGTCAATATCGGTTTTTAAGGTGTGTAAATC
CCTATTATTGATGCCGATAATGTCGTATTGGAGCTTGAGCAAGCGCTTGATTTCTTGCTTAT
TGGAAACTTCAGTCAGCACGCTCATGCTTAAGGATTTAGCGAGGTTGAAAAGCTCTAAAT
AATTCTTATCATCTAATACGCTTAACATTAAAAGCACCGCATTAGCCCCCATCATTCTAGCG
AGTTTGATTTGAAAGGCATCAATGATAAAATCTTTGCATAAAATGGGCTTAATGGAATGTT
GCGCAACCATCTTAATGTTTTCATAAGAGCCTAAAAAATATTTAGAATCGGCTAAAACTGA
AATGCACACCCAGTGTTGGATAAAGAGTAACCAAGCAGGGATCCAAGCGGTTAAAATGC
CCTCAATGATAGCAAGCCATGGAGTGAATTTCCCTAAAGGGATTTTTAAGATGTTTTCAAT
GAAAGCGGTAAGCCACAAAACACCCCAAGCCAACCAAATGATCGCCCACCAATCGCCTT
CAGTGATGCCTAACACTTTGTGGTCATCAAGCATATCGCTATAGTGGGATAAAATCGCAGC
AGGAACAGTGTTGATCGCTACGAATAAGCTATACCAAGAATAAGGCCTCCAATCCAAACC
AAAAGTGTGGTTGATAGCCGCATACAAGTAGGTAAAACCAAACAATAACCCAGTCGCTG
GCCCATAGAAACTAGTCAAATGGTGCGATACTTGAGCAATATCTTCGGCACCTTCTACAG

GGGCTGTAGGGTGGAGTGCAGAATAAGTGATGACAACTATATTACAAATAATGGAGAGCC
CACCCACAAAAAAGTTCATCACCGCAGTGCTTTTAGGATCGACTTTGGTTAACCCGCAAA
TCCCATTGCTGATTAAAACAATCCCAAGCTGGCTTTTCTCCCGGCTCCAGAACCCACACG
ATTATTGATTTCTAAAAAATTATCAATTGAGATATTTTTAGCGTTGAAATCCACTCTCGTGG
TGCGATCAGCGCTATCTTTAAAGCTTGTGAAAGAATTGCCATTGAATTGGCCCAAACGCA
AAGTGGCTTTTTGCATATTGACATCTAAATCCCCACCAGTGTAGTTTCTTAGCCCTGAAAG
TTTATAAGTCCCTACAGCGTCTTTCATATCCACTTCAAGCTTGTTCCATTGCCCGCCTTTGA
CCCAATAATGCCTAGCAGCATTCCCCCAATCCCAACCTCCATCAATCTTACTAGATAAAAG
GGATTTGTATAAGTCGTATTCCTTGTTAGGGAATTCATTAAAGCCTCTTCCTGCTTGAATG
CGCCAAACTTTATCGGGTTTATCCGGGGTTTTATTCGCTTCTTCAATACAAGCCATAAAAC
TTTAAAAACCATTGCGATTTTAGGCCAGCCTAATGTGGGGAAAAGCTCGTTATTTAACCG
CCTGGCTAGAGAAAGGATCGCTATCACTTCAGATTTTGCAGGCACTACACGAGACATTAA
TAAACGAAAAATCGCATTGAATGGCCATGAAGTGGAATTGCTAGATACAGGGGGCATGGC
TAAAGACGCTCTTTTGTCTAAAGAAATCAAAGCCCTTAATTTAAAAGCCGCTCAAATGAG
CGATTTGATTTTATATGTTGTAGATGGCAAGTCTATACCTAGCGATGAAGATCTTAAGCTTT
TTAGAGAGGTTTTTAAAACCAACCCTAACTGCTTTTTAGTGATCAATAAAATTGATAACGA
CAAAGAAAAAGAGCGAGCTTATGCGTTTTCTTCTTTTGGCATGCCAAAGAGTTTTAATAT
TTCTGTTTCGCACAATAGAGGCATTAGTGCCCTAATTGATGCGATATTGAACGCGCTAAAT
TTAAATCAAATCATAGAGCAGGATTTGGAT
>OK113
TCCGTTTCTAAGAAAATTTGCCCGTCTGTAATAGAAATGATATTCGTAGGGATATAAGCTG
AAACATCGCCCGCTTGAGTTTCCACAATAGGGAGTGCGGTCAAAGAGCCTGCACCCTTT
TCATCGCAAAGTTTAGCCGCTCTTTCTAAAAGCCGTGAGTGGATATAAAACACATCTCCA
GGAAAAGCCTCCCTACCTGGGGGTCTTCTCAAAATCAAAGAAATTTCTCTGTAAGCGAC
AGCATGCTTACTCAAATCATCATAAATGATTAGGGCATGGCGGGCATGATCTCTAAAGTAT
TCCCCCATAGCCACACCTGAATAAGGGGCTAAATATTGCATCGCAGCTGAATCTGAAGCC
GAAGCGTTGATCACAACGCTGTATTCCATCGCTCCGTATTCTTCTAATTTGCGAACCACTT
GCGCGACAGTGGATTCTTTTTGCCCAATAGCCACATAGATACAGATCACATTTTGCCCTTT
TTGGTTAATGATCGCATCGATCGCTACGGTGGTTTTACCGGTTTGTTTATCCCCAATGATCA
ATTCCCTTTGCCCGCGCCCAATAGGCACCAACGCATCAATGGCTTTAATGCCTGTTTGCAA
AGGCTCATGCACCGATTTTCTGTCCATAATGCCCGGGGCTTTTTGCTCTATTAGGCTAAAT
TCATTCGTTTCTATCTCACCCTTGCCATCAATAGGCTCACCCAAAGCGTTCAACACACGCC
CCACAACCGCATC
GCAATTGGGATGAGTGAGCTCAAAAAGGGCTTGAAAATTGAATTGGGGGGTGTGCCTTA
TAGGATCGTAGAATACCAACATGTCAAGCCCGGCAAGGGTGCAGCTTTTGTGCGCGCAA
AAATCAAGTCGTTTTTAGATGGTAAGGTGATTGAGAAAACTTTCCATGCGGGGGATAAGT

GCGAAGAGCCTAATCTGGTTGAAAAAACGATGCAATACCTTTATCACGATGGCGATACAT
ACCAATTCATGGACATAGAGAGCTATGAGCAAATCGCCTTGAACGACTCTCAAGTGGGC
GAGGCTTCTAAATGGATGCTAGACGGCATGCAAGTGCAGGTTTTATTGCATAATGACAAG
GCGATTTCAGTGGATGTGCCGCAAGTTGTGGCTTTAAAAATTGTAGAAACAGCCCCTAAT
TTTAAGGGCGATACTTCAAGTGCGAGCAAAAAACCAGCGACTTTAGAAACCGGTGCGGT
CGTGCAAGTGCCTTTCCATGTTTTAGAGGGTGAGATCATTAAAGTCAATACTTACCTAAAC
AATAAGGGTTTAAAGGGCAAAGTGCGCATTTGGGTTTAGGGGAGCAGATTAAAGCCCCT
AGATCAATTAGGGCTTGGTTATGATTAAAGCTTTCATTAAGATTGAGAAAGTCATTCGCCT
TAATTTGTAAGTCTTTAGCCGTGATATTTGGATCCAAACCAAAAAGCCTTAAAAGCACTC
GTTTGATGTTAGCATCCACGCATGCGCTCTTTTCTCTAAAGCCAAAACATAAAATCGCATT
AGCCGTGTATGCACCAATCCCGGGGAGTTTCAACAGGCTTTGATAGTCATTGGGTAATTG
TGAGTTGTGTTCTTTAACGCAAATTTCAGCGCTTTTTTTAAATTTTAGCCCTTGAATAAT
AACCAAGCCCTCTCCAGAGCAATAAAACCTCTTCTAATTGAGCGTTCGCTAAGTCTTTTA
AAGTGGGGAAAGCTTCTAAAAAAGGGGAATAAAAACGCTCAATTACCGTGTTGATTTGG
GTTTGTTGGCTCATCACTTCGCTGATATACTTTGTGCGTGGTGATTGAAATATCCAAGCATT
CTAATATCAAGTATGAATTGGATAAAGAAAGTGGGGCTTTAATGGTGGATAGGGTGCTTTA
TGGGGCGCAAAATTACCCCGCCAATTATGGCTTTGTGCCTAACACTCTAGGATCTGATGG
CGACCCTGTAGATGCGCTTGTTTTAAGCGATGTGGCTTTTCAAGCCGGGAGCGTAGTGAA
AGCGCGCTTGGTTGGGGTTTTGAACATGGAAGATGAAAGCGGAATGGATGAAAAACTGC
TCGCTCTACCCATAGATAAGATCGATCCCACGCATTCCTATGTCAAAGATATTGATGATTTA
TCCAAACACACTTTAGATAAAATCAAGCATTTTTTTGAAACTTACAAGGATTTAGAGCCTA
ATAAATGGGTGAAAGTCAAGGGGTTTGAAAACAAGGAGA
TTATTTTCGCCTAAAATCAATTTAATACACGCTTTTTTCAAATCCTTTTCTTTCATTAAAGA
GCTGCCCACTAAAAAGCCATTCACATAAGGGGCTAGAGTTTTGATTTGTGCGTGCGAATA
AATACCGGACTCGCTGATAATGAGCGCGTCTTTAGGCAAAAGGGGGCGTAATTTGAGCGT
GTGGTCAATATCGGTTTTTAAGGTGTGTAAATCCCTGTTATTGATGCCGATAATGTCGTATT
GGAGTTTGAGCAAGCGCTTGATTTCTTGCTGGTTAGAAACTTCAGTCAGCACGCTCATGT
TTAAGGATTTAGCGAGGTTGAAAAGCTCTAAATAATTTTTATCATCTAATACGCTTAACATT
AAAAGCACTGCATTAGCCCCCATCATTCTAGCGAGTTTGATTTGAAAAGCGTCAATGATA
AAATCTTTGCATAAAATAGGCTTAATGGAATGTTGCGAAACCATCTTAATGTTTTCATAAG
AGCCTAAAAAATATTTAGAATCGGCTAAAACCGAAATACAAGAGGCGAATTTTTCATAAG
TTTTGGCTATTTTCAACAGATCAAAATCTTTTCTGATCAAACCTTTAGAGGGCGATGCTTC
CCTTAGATTGCCTATTAAACGCTCTATGATCATCTCACACCCAGTGTTGGATAAAGAGTAA
CCAAGCGGGGATCCAAGCGGTTAAAATACCCTCAATGATAGCAAGCCATGGAGTGAATTT
CCCTAAAGGAATTTTCAAGATGTTTTCAATGAAAGCGGTAAGCCATAAAACACCCCAAGC
CAACCAAATGATCGCCCACCAATCGCCTTCAGTGATGCCTAACACTTTGTGGTCATCAAG

CATATCGCTATAGTGGGATAAAATCGCAGCAGGAACAGTGTTGATCGCTACGAATAAGCT
ATACCAAGAGTAGGGCCTCCAATCCAAACCAAAAGTGTGGTTGATAGCCGCATACAAGTA
GGTAAAACCGAACAATAACCCAGTCGCTGGCCCATAGAAACTGGTCAAATGGTGCGATA
CTTGAGCAATATCTTCTGCGCCTTCTACAGGGGCTGTAGGGTGGAGAGCAGAATAGGTGA
TGACAACCACATTACAAACAATGGAAAGCCCGCCCACAAAAAAGTTCATCACCGCAGTG
CTTTTAGGATCGACTTTGGTTAATCCGCAAATCCCGTTGCTGATTAAAACAATCCCAACAT
ATAACAATACAAGTCCTAGCATTGCCTTATCCTTCCAAAACAAAATTTTTACAACAAACGC
AGAAGAAGCGAATAAACCCCGGATAAACCCGATAAAGTTTGGCGCATTCAAGCAGGAAG
AGGTTTTAATGAATTCCCTAACAAGGAATACGACTTATACAAATCCCTTTTATCCAGTAAG
ATTGATGGAGGCTGGGATTGGGGGAATGCCGCTAGGCATTATTGGGTCAAAGGCGGGCA
ATGGAACAAGCTTGAAGTGGATATGAAAGATGCTGTAGGGACTTATAAGCTTTCAGGGCT
AAGAAACTTTACCGGTGGGGATTTGGATGTCAATATGCAAAAAGCCACTTTGCGTTTGGG
CCAATTCAATGGCAATTCTTTCACAAGCTTTAAGGATAGTGCTGATCGCACCACGAGAGT
GGATTTCAACGCTAAAAATATCTCAATTGATAATTTTTTAGAAATCAATAACCGCGTGGGT
TCTGGAGCCGGTAGGAAAGCTAGC
AATGCAAGCCATAAAACTTTAAAAACCATTGCGATTTTAGGCCAGCCTAATGTGGGGAAA
AGCTCGTTATTTAACCGCTTAGCTAGAGAAAGGATCGCTATCACTTCAGATTTTGCAGGCA
CTACACGAGACATTAACAAACGAAAAATCGCATTGAATGGCCATGAAGTGGAATTACTAG
ATACAGGGGGCATGGCTAAAGACGCTCTTTTGTCTAAAGAAATCAAAGCCCTTAATTTAA
AAGCCGCTCAAATGAGCGATTTGATTTTATATGTTGTAGATGGCAAGTCTATCCCTAGCGA
TGAAGATCTTAAGCTTTTTAGAGAAGTTTTTAAAACCAACCCTAACTGCTTTTTAGTGATC
AATAAAATTGACAACGACAAAGAAAAAGAGCGAGCTTATGCGTTTTCTTCTTTTGGCATG
CCAAAGAGTTTTAATATTTCCGCTTCGCACAATAGAGGCATTAGCGCCCTAATTGATGCGA
TATTGAACGCGCTAAATTTAAATCAAATCATAGAGCAGGATTTGGAT
>No1 Oral
GNCGCCGNNTTCATGCTTGGCTTG----
CANATGATCTTCGATATTTTGCTGTTTCTGGCATGCTGTTCCAGGAGAAGCACATCCTTGT
GGGGTTTGGGGAGTCTTCCCTCACTCCCCTCTCCTACGCGTAGACTATGCTACTTGAGAC
CAATCTTGACCTCCATCGTGGGGAACGATGTGACGTGCCCCGACGCGGGATTCCTGCTCT
-
TGCGAATCGATATGAAGATTCGGGTTTATGACGGCTCTGTCATTTAAGTTCCCCTGAATTT
TTGNANAAAGTGGATGAGCTTCTTCTTGACCTCCTCCAATCTCTGCTTGCTCATCTGAGG
AACT--
TCCNTTTTCTCNCGTCTAAAGCCGAATCCTTCATCACTCATCACTACGTATGGAGGGACTG
GCCCTATGAAACTGCTCCAACCGTTGGATGAGGTCAAAATTCTGCCTTTTGGAGCTTCTA
TTGCCTATGAGATGGGAGATTCNGAAGACTCTCTTTTCACCCNANAAAAGATCTTTTTCN

ANNNATGANGGNTGCTGNNAATCNGCTTCTGCTGAAGANTTTT----
TTCCNGAACATTTCATGAAAGCGGCGTC-----
TTTCNTCNNGANCNNCTGGGANTGCCGCACCTTACGAAATAACCTA
AAAGTGAAACTACAAGTAAAATGAATACAAGCCATAAAACTTTAAAAACCATTGCGATTT
TAGGCCAGCCTAATGTGGGGAAAAGCTCGTTATTTAACCGCTTGGCTAGAGAAAGGATCG
CTATCACTTCAGATTTTGCAGGCACTACACGAGACATTAACAAACGAAAAATCGCATTGA
ATGGTCATGAAGTGGAATTGCTAGATACAGGGGGCATGGCTAAAGGCGCTCTTTTGTCTA
AAGAAATCAAAGCCCTTAATTTAAAAGCCGCTCAAATGAGCGATTTGATTTTATACGTTGT
GGATGGCAAGTCTATCCCTAGCGATGAAGATCTTAAGCTTTTTAGAGAGGTTTTTAAAAC
CAACCCTAACTGCTTTTTAGTGATCAATAAAATTGATAACGACAAAGAAAAAGAGCGAG
CTTATGCGTTTTCTTCTTTTGGCATGCCCAAGAGTTTTAACATTTCCGTTTCGCACAATAG
AGGCATTAACGCCTTAATTGATGCAATATTGAGGGCGCTGGATTTAAATCAAATCATAGAG
CAGGATTTTGGATGCGGATATTTTAGAAAGCCTAGAAAATAACGCACCAGAAGAAGAAA
CTAAAGA
>No1 Gastric
TTNNGGTTNTTTTNNNNNNTGCGTTTGTTGTAAAAATTTTGTTTTGGAAGGATAAGGCAA
TGCTAGGACTTGTATTGTTATATGTTGGGATTGTTTTAATCAGCAACGGGATTTGCGGATTA
ACCAAAGTCGATCCTAAAAGCACTGCGGTGATGAACTTTTTTGTGGGTGGGCTCTCCATT
ATTTGTAATGTGGTTGTCATCACTTATTCTGCGCTCAACCCTACAGCCCCTGTAGAAGGCG
CAGAAGATATTGCTCAAGTATCGCACCATTTGACTAATTTCTATGGGCCAGCAACTGGGTT
ATTGTTCGGTTTTACCTACTTGTATGCGGCCATCAACCACACTTTTGGTTTGGATTGGAGG
CCCTACTCTTGGTATAGCTTATTCGTAGCGATCAACACTGTTCCTGCTGCGATTTTATCCCA
CTATAGCGATATGCTTGATGACCACAAAGTGTTAGGCATCACTGAAGGCGATTGGTGGGC
GATCATTTGGTTGGCTTGGGGTGTTTTGTGGCTTACCGCTTTCATTGAAAACATCTTGAAA
ATCCCTTTAGGGAAATTCACTCCATGGCTTGCTATCATTGAGGGTATTTTAACCGCTTGGA
TCCCTGCTTGGTTGCTCTTTATCCAACACTGGGTGTGAGATGATCATAGAGCGTTTAATAG
GCAATCTAAGGAAATTTA
TTTGATTAAAGTGAAACTACAAGTAAAATGAATACAAGCCATAAAACTTTAAAAACCATT
GCGATTTTAGGCCAACCTAATGTGGGGAAAAGCTCGTTATTTAACCGCTTAGCTAGAGAA
AGGATCGCTATCACTTCAGATTTTGCAGGCACTACACGAGACATTAACAAACGAAAAATC
GCATTGAATGGCCATGAAGTGGAATTGCTAGATACAGGGGGCATGGCTAAAGACGCTCTT
TTGTCTAAAGAAATCAAAGCCCTCAATTTAAAAGCCGCTCAAATGAGCGATTTGATTTTAT
ATGTTGTGGATGGCAAGTCTATCCCTAGCGATGAAGATCTTAAGCTTTTTAGAGAGGTTTT
TAAAACCAACCCTAACTGCTTTTTAGTGATCAATAAAATTGATAACGACAAAGAAAAAGA
GCGAGCTTATGCGTTTTCTTCTTTTGGCATGCCAAAGAGTTTTAACATTTCCGTTTCGCAC
AATAGAGGCATTAGTGCCTTAATTGATGCGATATTGAACGCGCTAAATTTAAATCAAATCA

TAGAGCAGGATTTGGATGCGGATATTTTAGAAAGCCTAGAAAATAACGCATCAAAAGA
>No2 Oral
ATTGAATTGGGCGGTGTGCCTTATAGGATCGTAGAATACCAACATGTCAAGCCCGGCAAG
GGTGCGGCTTTTGTGCGCACGAAAATCAAGTTGTTTTTAGATGGTAAGGTGATTGAAAAG
ACTTTCCATGCGGGGGATAAGTGCGAAGAGCCTAATCTGGTTGAAAAAACGATGCAATAC
CTTTATCACGATGGCGATACATACCAATTCATGGATATAGAGAGCTATGAGCAAATCGCCT
TGAACGACTCTCAAGTGGGCGAGGCTTCTAAATGGATGCTAGACGGCATGCAAGTGCAG
GTTTTATTGCATAATGACAAGGCGATTTCAGTGGATGTGCCGCAAGTTGTGGCTTTAAAG
ATTGTAGAAACAGCCCCTAATTTTAAGGGCGATACTTCAAGTGCGAGCAAAAAACCAGC
GACTTTAGAAACCGGTGCGGTTGTGCAAGTGCCTTTCCATGTTTTAGAGGGTGAGATCAT
TA
GCGCGGTCGANCNGTTCGTCAAGCGGGCATCCT-
GTATGCGCCGGGCAAAGCCTCCAACGCCGGCGGCGTGGCAACTTCAGGCTTGGA-
AATGAGCCAAAACGCCGTCCGTCTGGCTTGGCACCGNAAAAAACTGTACCAACGCTTGT
TCNACATCGTGATAAAAATCGNCGAATCATGCCTGANATTCGGCACTGACAAAACNACCT
TTCCACTACCTCAACGGTGCGCAACATCGACGGTTTCNTGCCTTGCCCCATGCGATGCTG
GCGCACTGTGATGAGTAAGTCTAAGTGCCCTGACTTTACGATGTGGAAATGTCCGTTTTC
AGTGCCGTATTTCA-
GGCAGGATTCGTGGATGCTTTGCATGATGTCGAACAAGCGTTGGTCGACTTCTTCGCGGC
TCCAAGCCAGACGGACGGCGTTTTGGCTCATTTCCAAGCCTGAAGTTGCCACGCCGCCG
GCGTTGGAGGCTTTGCCCGGCGCATACAGGATGCCCGCTTTGACGAACTGATCGACCGC
GCCCAAAGTAGAAGGCATGTTCGCACCTTACGAATTACC
>No2 Gastric
TGAATTGGGCGGTGTGCCTTATAGGATCGTAGAATACCAACATGTCAAGCCCGGCAAGGG
TGCGGCTTTTGTGCGCACGAAAATCAAGTCGTTTTTAGATGGTAAGGTGATTGAAAAGAC
TTTCCATGCGGGGGATAAGTGCGAAGAGCCTAATCTGGTTGAAAAAACGATGCAATACCT
TTATCACGATGGCGATACATACCAATTCATGGATATAGAGAGCTATGAGCAAATCGCCTTG
AACGACTCTCAAGTGGGCGAGGCTTCTAAATGGATGCTAGACGGCATGCAAGTGCAGGT
TTTATTGCATAATGACAAGGCGATTTCAGTGGATGTGCCGCAAGTTGTGGCTTTAAAGATT
GTAGAAACAGCCCCTAATTTTAAGGGCGATACTTCAAGCGCGAGCAAAAAACCAGCGAC
TTTAGAAACCGGTGCGGTTGTGCAAGTGCCTTTCCATGTTTTAGAGGGTGAGATCATTAA
G
TTNGGGTTNTTTTNNNANNNGCGTTTGTTGTAAAAATTTTGTTTTGGAAGGATAAGGCAA
TGCTAGGACTTGTATTGTTATATGTTGGGATTGTTTTGATCAGCAACGGGATTTGCGGATTA
ACCAAAGTCGATCCTAAAAGCACTGCGGTGATGAACTTTTTTGTGGGTGGGCTTTCCATT
GTTTGTAATGTGGTTGTCATCACTTATTCTGCGCTCAACCCTACAGCCCCTGTAGAAGGTG

CAGAAGATATTGTTCAAGTATCGCACCATTTGACTAATTTCTATGGGCCAGCAACTGGGTT
ATTGTTCGGTTTTACCTACTTGTATGCGGCTATCAACCACACTTTTGGTTTGGATTGGAGG
CCCTACTCTTGGTATAGCTTATTCGTAGCGATCAACGCTGTTCCTGCTGCGATTTTATCCCA
CTATAGCGATATGCTTGATGACCACAAAGTGTTAGGCATCACTGAAGGCGATTGGTGGGC
GATCATTTGGTTGGCTTGGGGTGTTTTGTGGCTTACCGCTTTCATTGAAAACATCTTGAAA
ATCCCTTTAGGGAAATTCACTCCATGGCTTGCTATCATTGAGGGTATTTTAACCGCTTGGA
TCCCTGCTTGGTTACTCTTTATCCAACACTGGGTGTGAGATGATCATAGAGCGTTTAATAG
GCAATCTAAGGNNANTTTAAACA
>No3 Oral
GCGGTTGTGGGGCGTGTGTTGAACGCTTTGGGTGAGCCTATTGATGGTAAGGGTGAGATA
GAAACAAATGAATTTAGCCTCATCGAGCAAAAAGCCCCGGGCATTATGGACAGAAAATC
GGTGCATGAGCCTTTGCAAACAGGCATTAAAGCCATTGATGCGTTGGTGCCTATTGGGCG
CGGGCAAAGGGAATTGATCATTGGGGATAAACAAACCGGTAAAACCACCGTAGCGATCG
ATGCGATCATTAACCAAAAAGGGCAAAAATGTGATTTGTATCTATGTGGCTATTGGGCAA
AAAGAATCCACTGTCGCGCAAGTGGTCCGCAAATTAGAAGAATACGGAGCGATGGAATA
CAGCGTCGTGATCAACGCTTCGGCTTCAGATCCAGCTGCGATGCAATACTTAGCCCCTTAT
TCAGGTGTGGCTATGGGGGAATACTTTAGAGATCATGCCCGCCATGCCCTAATCATTTATG
ATGATTTGAGTAAGCATGCTGTCGCTTACAGAGAAATTTCTTTGATTTTGAGAAGACCCC
CAGGTAGGGAGGCTTTTCCTGGAGATGTGTTTTATATCCACTCACGGCTTTTAGAAAGAG
CGGCTAAACTTTGCGATGAAAAGGGTGCAGGCTCTTTGACCGCACTCCCTATTGTGGAA
ACTCAAGCGGGCGATGTTTCAGCTTATATCCCTACGAATATCATTTCTATTACAGACGGGC
AAATTTTCTTAGAAACGGATT
GAATTGGGCGGTGTGCCTTATAGGATCGTAGAATACCAGCATGTCAAGCCCGGCAAGGGT
GCGGCTTTTGTGCGCGCAAAAATCAAGTCGTTTTTAGATGGCAAGGTGATTGAAAAGAC
TTTCCATGCGGGGGATAAGTGCGAAGAGCCTAATCTGGTTGAAAAAACGATGCAATACCT
TTATCACGATGGAGATACATACCAATTCATGGACATAGAGAGCTATGAGCAAATCGCCTTA
AACGACTCTCAAGTGGGCGAGGCTTCTAAATGGATGCTAGACGGCATGCAAGTGCAGGT
TTTATTGCATAATGACAAGGCGATTTCAGTGGATGTGCCGCAAGTTGTGGCTTTAAAAATT
GTAGAAACAGCCCCTAATTTTAAGGGCGATACTTCAAGTGCGAGCAAAAAACCAGCGAC
TTTAGAAACCGGTGCGGTCGTGCAAGTGCCTTTCCATGTTTTAGAGGGTGAGATC
TTTTTTATATCAGTGAAGTGATGAGCCAACAAACCCAAATCAACACGGTAATTGAGCGTT
TTTATTCCCCTTTTTTAAAAGCTTTCCCCACTTTAAAAGACTTAGCGAACGCTCCATTAGA
GGAGGTTTTATTGCTCTGGAGAGGGCTTGGCTATTATTCAAGGGCTAAAAATTAAAAAA
AAGCGCTGAAATTTGCGTTAAAGAACACAACTCGCAATTACCCAATGACTATCAAAGCCT
GTTGAAACTCCCTGGGATTGGTGCATACACGGCTAATGCGATTTTATGTTTTGGCTTTAGA
GAAAAGAGCGCATGCGTGGATGCTAATATCAAACGAGTGCTTTTAAGGCTTTTTGGTTTG

GATCCAAATATCACAGCTAAAGATCTACAAATTAAGGCGAATGACTTTCTCAATCTTAATG
AAAGTTTTAATCATAACCAAGCCCTAATTGATCTAGGGGCTTTAATCTGCTCCCCTAAACC
CAAATGTGCACTTTGCCCTTTCAATCCTTATTGGTTTAGGTAA
AGCCTTTGTGCGTGGTGATTGAAATATCCAAGCATTCTAATATCAAGTATGAACTGGATAA
AGAAAGCGGGGCTTTAATGGTGGATAGGGTGCTTTATGGGGCACAAAATTACCCCGCCA
ATTATGGCTTTGTGCCTAACACTTTAGGATCTGATGGCGACCCTGTAGATGCGCTTGTTTT
AAGCGATGTGGCTTTTCAAGCCGGGAGCGTGGTGAAAGCGCGCTTGGTTGGGGTTTTGA
ACATGGAAGATGAAAGCGGAATGGATGAAAAATTACTCGCTCTACCCATAGATAAGATCG
ATCCCACGCATTCCTATGTCAAAGATATTGATGATTTATCCAAACACACTTTAGATAAAATC
AAGCATTTTTTTGAAACTTACAAGGATTTAGAGCCTAATAAATGGGTGAAAGTCAAGGGG
TTTGAA
TAGCTTCGCCCCTNTAAAGGTTTAATCAGAAAAGATTTTGATCTGTTGAAAATAACCAAA
ACTTATGAAAAATTCGCCTCTTGTATTTCGGTTTTAGCCGATTCTAAATATTTTTTAGGCTC
TTATGAAAACATTAAGATGGTTTCGCAACATTCCATTAAGCCCATTTTATGCAAAGATTTTA
TCATTGACGCTTTTCAAATCAAACTCGCTAGAATGATGGGGGCTAATGCAGTGCTTTTAAT
GTTAAGCGTATTAGATGATAAAAATTATTTAGAGCTTTTCAACCTCGCTAAATCCTTAAAC
ATGAGCGTGCTGACTGAAGTTTCTAACCAGCAAGAAATCAAGCGCTTGCTCAAACTCCA
ATACGACATTATCGGCATCAATAACAGGGATTTACACACCTTAAAAACCGATATTGACCAC
ACGCTCAAATTACGCCCCCTTTTGCCTAAAGACGCACTCATTATCAGCGAGTCCGGTATTT
ATTCGCACGCACAAATCAAAGCCCTAGCCCCTTATGTGAATGGCTTTTTAGTGGGCAGCT
CTTTAATGAAAGAAAGGGATTTGAAAAAAGCGTGTATTAAATTGATTTTAGGCGAAAATA
A
GCGTTTGTTGTAAAAATTTTGTTTTGGAAGGATAAGGCAATGCTAGGACTTGTATTGTTAT
ATGTTGGGATTGTTTTAATCAGCAACGGGATTTGCGGATTAACCAAAGTCGATCCTAAAA
GCACTGCGGTGATGAACTTTTTTGTGGGTGGGCTCTCCATTATTTGTAATGTGGTTGTCAT
CACTTATTCTGCACTCAACCCTACAGCCCCTGTAGAAGGCACAGAAGATATTGCTCAAGT
ATCGCACCATTTGACTAATTTCTATGGGCCAGCAACTGGGTTATTGTTCGGTTTTACCTACT
TGTATGCGGCCATCAACCACACTTTTGGTTTGGATTGGAGGCCCTATTCTTGGTATAGCTT
ATTCGTAGCAATCAACACTGTTCCTGCTGCGATTTTATCCCACTATAGCGATATGCTTGATG
ACCACAAAGTGTTAGGCATCACTGAAGGCGATTGGTGGGCGATCATTTGGTTGGCTTGGG
GTGTTTTGTGGCTTACCGCTTTCATTGAAAACATCTTGAAAATCCCTTTAGGGAAATTCAC
TCCATGGCTTGCTATCATTGAGGGTATTTTAACCGCTTGGATCCCTGCTTGGTTGCTCTTTA
TCCAACACTGGGTGTGAGATGATCATAGAGCGTTTAATAGGCAATCTAAGGGNANTTTAA
ANA
CTTTACAACCGTGATCATTCCAGCCATTGTTGGAGGTATCGCTACAGNNTCTGCTGTAGG
GACGGTCTCAGGGCTTCTTGGTTGGGGACTCAAACA-

AGCCGAAGAAGCTAATAAAACCCCGGATAAACCCGATAAAGTTTGGCGCATTCAAGCAG
GAAGAGGTTTTAATGAATTCCCTAACAAGGAATACGACTTATACAAATCCCTTTTATCCAG
TAAGATTGATGGAGGCTGGGATTGGGGGAATGCCACTAGGCATTATTGGGTCAAAGGCGG
GCAATGGAACAAGCTTGAAGTGGATATGAAAGACGCTGTAGGGACTTATAAGCTTTCAG
GGCTAAGAAACTTTACCGGTGGGGATTTAGATGTCAATATGCAAAAAGCCACTTTGCGTT
TGGGTCAATTCAATGGCAATTCTTTCACAAGCTTTAAGGATAGCGCTGATCGCACCACAA
GAGTGGATTTCAATGCTAAAAATATCTTAATTGATAATTTTTTAGAAATCAATAATCGTGTG
GGTTCTGGAGCTGGGAGGAAAGCCAGCTCTACGGTTTTGACTTTGCAAGCTTCAGAAGG
GATCACTAGCGGTAAAAACGCTGAAATTTCTCTTTATGANGNNGCCCACGCAN
TGAAACTACAAGTAAAATGAATACAAGCCATAAAACTTTAAAAACCATTGCGATTTTAGG
CCAGCCTAACGTGGGGAAAAGCTCGTTATTTAACCGCTTAGCTAGAGAAAGGATCGCTAT
CACTTCAGATTTTGCAGGCACTACACGAGACATTAACAAACGAAAAATTGCATTGAATGG
TCATGAAGTGGAATTGCTAGATACAGGGGGCATGGCTAAAGACGCTCTTTTGTCTAAAGA
AATCAAAGCCCTTAATTTAAAAGCCGCTCAAATGAGCGATTTGATTTTATATGTTGTGGAT
GGTAAGTCTATCCCTAGCGATGAAGATCTTAAGCTTTTTAGAGAGGTTTTTAAAACCAAC
CCTAACTGCTTTTTAGTGATCAATAAAATTGATAACGACAAAGAAAAAGAGCGAGCTTAT
GCGTTTTCTTCTTTTGGCATGCCAAAGAGTTTTAATATTTCCGTTTCGCACAATAGAGGCA
TTAGCGCCTTAATTGATGCAATATTGAACGCACTAAATTTAAATCAAATCATAGAGCAGGA
TTTGGATGCGGATATTTTAGAAAGCCTAGAAAATAACGCACCAAAAGAAGA
>No3 Gastric
GATGCGGTTGTGGGGCGCGTGTTGAACGCTTTGGGTGAGCCTATTGATGGCAAGGGTGA
GATAGAAACGAATGAATTTAGTCTCATCGAGCAAAAAGCCCCAGGCATTATGGACAGAA
AATCGGTGCATGAGCCTTTGCAAACAGGCATTAAAGCCATTGATGCGTTGGTGCCTATTG
GGCGCGGGCAAAGGGAATTGATCATTGGGGACAAACAAACCGGTAAAACCACTGTAGC
GATCGATGCGATCATTAACCAAAAAGGGCAAAATGTGATCTGTATCTATGTGGCTATTGGG
CAAAAAGAATCCACTGTCGCACAAGTGGTCCGCAAATTAGAAGAATACGGAGCGATGGA
ATACAGTGTTGTGATCAACGCTTCGGCTTCAGATTCGGCTGCGATGCAATATTTAGCCCCT
TATTCAGGTGTGGCTATGGGGGAATACTTTAGAGATCATGCCCGCCATGCCCTAATCATTT
ATGATGATTTGAGTAAGCATGCTGTCGCTTACAGAGAAATTTCTTTGATTTTGAGAAGACC
TCCAGGTAGGGAGGCTTTTCCTGGAGATGTGTTTTATATCCACTCACGGCTTTTAGAAAG
AGCGGCTAAACTTTGCGATGAAAAGGGTGCAGGCTCTTTGACCGCGCTCCCTATTGTGG
AAACTCAAGCGGGCGATGTTTCAGCCTATATCCCTACAAATATCATTTCTATTACAGACGG
ACAAATTTTCTTAGAAACGGA
GCAATTTGGATGAGCGAGCTCAAAATGGGTTTGAAAATTGAATTGGGGGGTGTGCCTTAT
AGGATCGTAGAATACCAACATGTCAAGCCCGGCAAGGGCGCGGCTTTTGTGCGTGCGAA
AATCAAGTCGTTTTTAGATGGTAAGGTGATTGAGAAGACTTTCCATGCGGGGGATAAGTG

CGAAGAGCCTAATTTAGTTGAAAAAACGATGCAATACCTTTATCATGATGGCGATACATAC
CAATTCATGGATATAGAGAGCTATGAGCAAATCGCTTTGAACGACTCTCAAGTGGGCGAG
GCTTCTAAATGGATGTTAGACGGCATGCAAGTGCAGGTTTTATTGCATAATGACAAGGCG
ATTTCAGTGGATGTGCCGCAAGTTGTGGCTTTAAAAATTGTAGAAACAGCCCCTAATTTT
AAGGGCGATACTTCAAGCGCGAGCAAAAAACCAGCGACTTTAGAAACCGGTGCGGTCG
TGCAAGTGCCTTTCCATGTTTTAGAGGGTGAGATCATTAAGTCAATACA

TATATCAGCGAAGTGATGAGCCAACAAACCCAAATCAACACGGTAGTTGAGCGTTTTTAT
TCCCCTTTTTTAAAAGCTTTCCCCACTTTAAAAGACTTAGCGAACGCTCCATTAGAGGAG
GTTTTATTGCTCTGGAGAGGGCTTGGCTATTATTCAAGGGCTAAAAATTTAAAAAAAAGC
GCTGAAATTTGCGTTAAAGAACACAACTCGCAATTACCCAATGACTATCAAAGCCTGTTG
AAACTCCCTGGGATTGGTGCATACACGGCTAATGCGATTTTATGTTTTGGCTTTAGAGAAA
AGAGCGCATGCGTGGATGCTAATATCAAACGAGTGCTTTTAAGGCTTTTTGGTTTGGATCC
AAATATCACAGCTAAAGATCTACAAATTAAGGCGAATGACTTTCTCAATCTTAATGAAAGT
TTTAATCATAACCAAGCCCTAATTGATCTAGGGGCTTTAATCTGCTCCCCTAAACCCAAAT
GCACGATTTGCCCTTTCAATCCTTATTGTTTAGGTAA

TGAGTCTTTGTGCGTGGTGATTGAAATATCCAAGCATTCTAATATCAAGTATGAACTGGAT
AAAGAAAGCGGGGCTTTAATGGTGGATAGGGTGCTTTATGGGGCGCAAAATTACCCCGC
AAATTATGGCTTTGTGCCTAACACTTTAGGATCTGATGGCGACCCTGTAGATGCGCTTGTT
TTAAGCGATGTGGCTTTTCAAGCCGGGAGCGTGGTGAAAGCGCGCTTGGTTGGGGTTTT
GAACATGGAAGATGAAAGCGGAATGGATGAAAAATTACTCGCTCTGCCCATAGATAAGAT
TGATCCCACGCATTCCTATGTCAAAGACATTGATGATTTATCCAAACACACTTTAGATAAA
ATCAAGCATTTTTTTGAAACTTACAAGGATTTAGAACCTAATAAATGGGTGAAAGTCAAG
GGGTTTGAAAACAAAGAGA

TAGCATCGGCCCTCTAAAGGTTTAATAAGAAAAGATTTTGATCTATTGAAAATAGCCAAG
ACTTATGAAAGATTCGCCTCTTGCATTTCAGTTTTAGCCGATTCTAAATATTTTTTAGGCTC
TTATGAAAACATTAAGATGGTTTCGCAACATTCCATTAAGCCCATTTTATGCAAAGATTTTA
TCATTGACGCTTTTCAGATCAAACTCGCTAGAATGATGGGGGCTAATGCGGTGCTTTTAAT
GTTAAGCGTATTAGATGATAAAAATTATTTAGAGCTTTTCAACCTCGCTAAATCCTTAAAC
ATGAGCGTGCTGACTGAAGTTTCTAACCAGCAAGAAATCAAGCGCTTGCTCAAACTCCA
ATACGACATTATCGGCATCAATAACAGGGATTTACACACCTTAAAAACCGATATTGACCAC
ACGCTCAAATTACGCCCCCTTTTGCCTAAAGACACGCTCATTATCAGCGAGTCCGGTATTT
ATTCGCACGCGCAAATCAAAGCCCTAGCCCCTTATGTGAATGGCTTTTTAGTGGGCAGCT
CTTTAATGAAAGAAAAGGGATTTGAAAAAAGCGTGTATTAAATTGATTTTAGGCGAAAAT
AATGGGGGGGGGG

TTATTTNNNNNNTGCGTTTGTTGTAAAAATTTTGTTTTGGAAGGATAAGGCAATGCTAGG
ACTTGTATTGTTGTATGTTGGGATTGTTTTAATCAGCAATGGGATTTGCGGATTAACCAAA

GTCGATCCTAAAAGCACTGCGGTGATGAACTTTTTTGTGGGTGGGCTTTCCATTGTTTGTA
ATGTGGTTGTCATCACCTATTCTGCTCTCCACCCTACAGCCCCTGTAGAAGGCGCAGAAG
ATATTGCTCAAGTATCGCACCATTTGACCAGTTTCTATGGGCCAGCGACTGGGTTATTGTT
CGGTTTTACCTACTTGTATGCGGCTATCAACCACACTTTTGGTTTGGATTGGAGGCCCTAC
TCTTGGTATAGCTTATTCGTAGCGATCAACACTGTTCCTGCTGCGATTTTATCCCACTATAG
CGATATGCTTGATGACCACAAAGTGTTAGGCATCACTGAAGGCGATTGGTGGGCGATCAT
TTGGTTGGCTTGGGGTGTTTTGTGGCTTACCGCTTTCATTGAAAACATCTTGAAAATCCCT
TTAGGGAAATTCACTCCATGGCTTGCTATCATTGAGGGTATTTTAACCGCTTGGATCCCTG
CTTGGTTACTCTTTATCCAACACTGGGTGTGAGATGATCATAGAGCGTTTAATAGGCAATC
TAANGGAAATTTA
AACCGNGNTNNNNNNCCAGCCATTGTTGGAGGTATCGCTTCAGGTGCTGCTGTNNNNNC
GGTCTCAGGGCTTCTTGGTTGGGGACTCAAACAAGCCGAAGAAGCTAATAAAACCCCGG
ATAAACCCGATAAAGTTTGGCGCATTCAAGCAGGAAGAGGTTTTAATGAATTCCCTAACA
AGGAATACGACTTATACAAATCCCTTTTATCCAGTAAGATTGATGGAGGCTGGGATTGGGG
GAATGCCACTAGGCATTATTGGGTCAAAGGCGGGCAATGGAACAAGCTTGAAGTGGATAT
GAAAGACGCTGTAGGGACTTATAAGCTTTCAGGGCTAAGAAACTTTACCGGTGGGGATTT
AGATGTCAATATGTAAAAAGCCACTTTGCGTTTGGGCCAATTCAATGGCAATTCTTTCACA
AGCTTTAAGGATAGCGCTGATCGCACCACGAGAGTGAATTTCAACGCTAAAAATATCTCA
ATTGATAATTTTTTAGAAATCAATAATCGTGTGGGTTCTGGAGCCGGGAGGAAAGCCAGC
TCTACGGTTTTAACTTTGCAAGCTTCAGAAGGGATCACTAGCGGTAAAAACGCTGAAATT
TCTCTTTATGATGGCGCCACGCATAATTTGGCTTCAAACAGCGTTAAATTAATGGGTAATG
TGTGGATGGGCCGTTTGCAATACGTGGGAAGCGTATAATCCCTATTGTGGAAACTCAAA
TGAAACTACAAGTAAAATGAATACAAGCCATAAAACTTTAAAAACCATTGCGATTTTAGG
CCAGCCTAATGTGGGGAAAAGCTCGTTATTTAACCGCTTGGCTAGAGAAAGGATCGCTAT
CACTTCAGATTTTGCAGGCACTACACGAGACATTAACAAACGAAAAATCGCATTGAATGG
TCATGAAGTGGAATTGCTAGATACAGGGGGCATGGCTAAAGACGCTCTTTTGTCTAAAGA
AATCAAAGCCCTTAATTTAAAAGCCGCTCAAATGAGCGATTTGATTTTATACGTTGTGGAT
GGCAAATCTATCCCTAGCGATGAAGATCTTAAGCTTTTTAGAGAGGTTTTTAAAACTAACC
CTAACTGCTTTTTAGTGATCAATAAAATTGATAACGACAAAGAAAAAGAGCGAGCTTATG
CGTTTTCTTCTTTTGGCATGCCGAAGAGTTTTAACATTTCCGTTTCGCACAATAGAGGCAT
TAGTGCCTTAATTGATGCGATATTGAGCACGCTAAATTTAAATCAAATCATAGAGCAAGAT
CTAGATGCGGATATTTTAGAAAGCCTAGAAAATAACGCACCAAAAGAAG
>N05 Oral
TAATCCTTTCCGCAAAATCTTCGGCAGTCAGGGTTTAGGTTTTGCTGTTTGCTCCCCCAAT
TTTTTTGCCGTTGGGCAGCCATTGGTAGCTGACTTTGCTTTCGTCAAAGTTATCAGCGTCA
TGGACTTACGGCGGTCAGGGTCTCACCGACCTTGGCGTTACCACTGATAGTTACGGTGCC

TTCGTGATTAGTCCCAGGCGGGGTTTGCCCGTCAGTGACAGCTTCAGTTTCCCCACTCTG
CGGGGTTTCATTGTGCTGTGCATTATCGGTGTAAGTTGCTTTGACACTGATTTTCGGGCCA
ATATCATTGACGGTCAGGGGGTAGGCCTTGCCGTTGGCGCCGGCGATTTCTTTGCCCTCG
GGCAGCCATTGGTAGCTGAGTTTTTTTCAACGAATAATCGTCGTGG
ATTAAAGTGAAACTACAAGTGAAATGAATACAAGCCATAAAACTTTAAAAACCATTGCGA
TTTTAGGCCAGCCTAATGTGGGGAAAAGCTCGTTATTTAACCGCTTAGCTAGAGAAAGGA
TCGCTATCACTTCAGATTTTGCAGGCACTACACGAGACATTAACAAACGAAAAATCGCAT
TGAATGGCCATGAAGTGGAATTGCTAGATACAGGGGGCATGGCTAAAGACGCTCTTTTGT
CTAAAGAAATCAAAGCCCTTAATTTAAAAGCCGCTCAAATGAGCGATTTGATTTTATATGT
TGTGGATGGCAAGTCTATCCCTAGCGATGAAGATCTTAAGCTTTTTAGAGAGGTTTTTAAG
ATCAACCCTAACTGCTTTTTAGTGATCAATAAAATTGATAACGACAAAGAAAAAGAGCGA
GCTTATGCGTTTTCTTCTTTTGGCATGCCAAAGAGTTTTAACATTTCCGTTTCGCACAATA
GAGGCATTAGCGCCTTAATTGATGCGATATTGAGCGCGCTAAATTTAAATCAAATCATAGA
GCAGGATTTGGATGCGGATATTTTAGAAAGCCTAGAAAATAACGCATCAAAAGAA
>No5 Gastric
AGTCTTTGTGCGTGGTGATTGAAATATCCAAGCATTCTAATATCAAGTATGAACTGGATAA
AGAAAGCGGGGCTTTAATGGTGGATAGGGTTCTTTATGGGGCGCAAAATTACCCCGCAAA
TTATGGCTTTGTGCCTAACACTTTAGGATCTGATGGCGACCCTGTAGATGTGCTTGTTTTA
AGCGATGTGGCTTTTCAAGCCGGGAGCGTGGTGAAAGCGCGCTTGGTTGGGGTTTTGAA
CATGGAAGATGAAAGCGGGATGGATGAAAAATTACTCGCTCTACCCATAGATAAGATCGA
TCCCACGCATTCCTATGTCAAAGATATTGATGATTTATCCAAACACACTTTAGATAAAATCA
AGCATTTTTTTGAAACTTACAAGGATTTAGAGCCTAATAAATGGGTGAAAGTCAAGGGGT
TT
TTTGATTAAAGTGAAACTACAAGTAAAATGAATACAAGCCATAAAACTTTAAAAACCATT
GCGATTTTAGGCCAGCCTAATGTGGGGAAAAGCTCGTTATTTAACCGCCTGGCTAGAGAA
AGGATCGCTATCACTTCAGATTTTGCAGGCACTACACGAGACATTAACAAACGAAAAATC
GCATTGAATGGCCATGAAGTGGAATTGCTAGATACAGGGGGCATGGCTAAAGACGCTCTT
TTGTCTAAAGAAATCAAAGCCCTTAATTTAAAAGCCGCTCAAATGAGCGATTTGATTTTAT
ATGTTGTGGATGGCAAGTCTATCCCTAGCGATGAAGATCTTAAGCTTTTTAGAGAGGTTTT
TAAAACCAACCCTAACTGCTTTTTAGTGATCAATAAAATTGACAACGACAAAGAAAAAG
AGCGAGCTTATGCGTTTTCTTCTTTTGGCATGCCAAAGAGTTTTAATATTTCCGTTTCGCA
CAATAGAGGCATTAGCGCCTTAATTGATGCGATATTGAACGCGCTAAATTTAAATCAAATC
ATAGAGCAGGATTTGGATGCGGATATTTTAGAAAGCCTAGAAAATAACGCAT
>No6 Oral
ANNNNNNGNNAAATTGAGCGTTTTTATTCCCCTTTTTTAGAAGCTTTCCCCACTTTAAAA
GACTTAGCGAACGC--

TCAATTAGAAGAGGTTTTATTGCTCTGGAGAGGGCTTGGCTACTATTCAAGGGCTAAAAA
TTTAAAAAAAAGCGCTGAAATTTGCATTAAAGAGCAAATGACTCACAATTA-
CCCAATGACTATCAAAGCCTGTTGAAACTCCCCGGGAT-
TGGTGCATACACGGCTAATGCAATTTTATGTTTTGGCTTTAGAGAAAAGAGAGCATGCGT
AGATGCTAATATCAAACGGGTGCTTTTAAGTCATGCTTTTTGGTTTGGATCCAAATATTCA
CGGCTAAAGACTTACAAATTAAATAGGCGAATGACTTTCTCAATCTTAA------
TGAAAGCTTTAATCATAACCAAGCCCTAATTGAT--
CTAGGGGCTTTAATCTGCTCCCCTAAACCCAGAATAAAAATGCGCACTTTGCCCTTTCAAT
CCTTATTGTTTAGGTAA
TTGCTTCGCCCTNTAAAGGTTTAATAAGAAAAGATTTTGATCTATTGAAAATAGCCAAGAC
TTATGAAAGATTCGCCTCTTGCATTTCAGTTTTAGCCGATTCTAAATATTTTTTAGGCTCTT
ATGAAAACATTAAGATGGTTTCGCAACATTCCATTAAGCCCATTTTATGCAAAGATTTTAT
CATNGACGCTTTTCAGATCAAACTCGCTAGAATGATGGGGGCTAATGCGGTGCTTTTAAT
GTTAAGCGTATTAGATGATAAAAATTATTTAGAGCTTTTCAACCTCGCTAAATCCTTAAAC
ATGAGCGTGCTGACTGAAGTTTCTAACCAGCAAGAAATCAAGCGCTTGCTCAAACTCCA
ATACGACATTATCGGCATCAATAACAGGGATTTACACACCTTAAAAACCGATATTGACCAC
ACGCTCAAATTACGCCCCCTTTTGCCTAAAGACACGCTCATTATCAGCGAGTCCGGTATTT
ATTCGCACGCGCAAATCCAAAGCCCTAGCCCCTTATGTGAATGGCTTTTTAGTGGGCAGC
TCTTTAATGAAAGAAAAGGATTTGAAAAAAGCGTGTATTAAATTGATTTTAGGCGAAAAT
AAAGTGTGCGGGCT
>No6 Gastric
TTNNTTNNNCTTTTTTTTATATCNNNNAAGTGATGAGCCANNAAACCCANCNTCNNNCAC
GGTANttGAGCGTTTTTATTCCCCTTTTTTAAAAGCTTTCCCCACTTTAAAAGACTTAGCGA
ATGCTCAATTAGAAGAGGTTTTATTGCTCTGGAGAGGGCTTGGCTATTATTCAAGGGATAA
AAATTTAAAAAAAAGCGCTGAAATTTGCGCTAAAGAACATAACTCACAATTACCCAATGA
CTATCAAAGCCTGTTGAAACTCCCTGGGATTGGTGCATACACGGCTAATGCGATTTTATGT
TTTGGCTTTAGAGAAAAGAGCGCATGCGTGGATGCGAACATCAAACGAACGCTTTTAAG
GCTTTTTGGTTTGAATCCAAATATCACGGCTAAAGATCTACAAATTAAGGCGAATGACTTT
CTCAATCTCAATGAAAGCTTTATCATAACCAAGCCCTAATTGATCTAGGGGCTTTAATCTG
CTCCCCTAAACCCAAATGTGCGGTTTGCCCTTTCAATCCTTATTGTTTAGGTAA
TCGCCCTCTAAAGGTTTAATCAGAAAAGATTTTGATCTGTTGAAAATAACCAAAACTTAT
GAAAAATTCGCCTCTTGCATTTCGGTTTTAGCCGATTCTAAATATTTTTTAGGCTCTTATAA
AAACATTAAGATGGTTTCGCAACATTCCATTAAGCCCATTTTATGCAAAGATTTTATCATTG
ATGCTTTTCAGATCAAACTCGCTAGAATGATGGGGGCTAATGCGGTGCTTTTAATGTTAAG
CGTATTAGATGATAAGAATTATTTAGAGCTTTTCAACCTCGCTAAATCCTTAAACATGAGC
GTGCTGACTGAAGTTTCCAACCAGCAAGAAATCAAGCGCTTGCTCAAACTCCAATACGA

CATTATCGGCATCAATAACAGGGATTTACACACCTTAAAAACCGATATTGACCACACGCTC
AAATTGCGCCCCCTTTTGCCTAAAGACGCGCTCATCATCAGCGAGTCCGGTATTTATTCGC
ATGCGCAAATCAAAGCCCTAGCCCCTTATGTGAATGGCTTTTTAGTGGGTAGCTCTTTAAT
GAAAGAAAAGGATTTGAAAAAAGCGTGTATTAAATTGATTTTAGGCGAAAATAAAGTG
>No8 Gastric
AGTCTTTGTGCGTGGTGATTGAAATATCCAAGCATTCTAATATCAAGTATGAATTGGATAA
AGAAAGCGGGGCTTTAATGGTGGATAGGGTGCTTTATGGGGCGCAAAATTACCCCGCCA
ATTATGGCTTTGTGCCTAACACTTTAGGATCTGATGGCGACCCTGTAGATGCGCTTGTTTT
AAGCGATGTGGCTTTTCAAGCCGGGAGCGTGGTGAAAGTGCGCTTGGTTGGGGTTTTGA
ACATGGAAGATGAAAGCGGAATGGATGAAAAATTACTCGCTCTACCCATAGATAAGATCG
ATCCCACGCATTCCTATGTCAAAGATATTGATGATTTATCCAAACACTACTTTAGATAAAAT
CAAGCATTTTTTTGAAACTTACAAGGATTTAGAGCCTAATAAATGGGTGAAAGTCAAGGG
GTT
TAGGTTATTNNTAAGGTGCGTTTGTTGTAAAAATTTGTTTTGGAAGGATAAGGCAATGCT
AGGACTTGTATTGTTATATGTTGGGATTGTTTTAATCAGCAACGGGATTTGCGGATTAACC
AAAGTCGATCCTAAAAGCACTGCGGTGATGAACTTTTTTGTGGGCGGGCTTTCCATTATT
TGTAATGTGGTTGTCATCACTTATTCTGCACTCAACCCTACAGCCCCTGTAGAAGGCGCA
GAAGATATTGCTCAAGTATCGCACCATTTGACTAATTTCTATGGGCCAGCGACTGGGTTAT
TGTTCGGTTTTACCTACTTGTATGCGGCTATCAACCACACTTTTGGTTTGGATTGGAGACC
CTACTCTTGGTATAGCTTATTCGTAGCGATCAACACTGTTCCTGCTGCGATTTTATCCCACT
ATAGCGATATGCTTGATGACCACAAAGTGTTAGGCATCACTGAAGGCGATTGGTGGGCGA
TCATTTGGTTGGCTTGGGGTGTTTTGTGGCTTACCGCTTTCATTGAAAACATCTTGAAAAT
CCCTTTAGGGAAATTCACTCCATGGCTTGCTATCATTGAGGGTATTTTAACCGCTTGGATT
CCTGCTTGGTTACTCTTTATCCAACACTGGGTGTGAGATGATCATAGAGCGTTTAACAGGC
AATCTAAGGAAATTTAAACANNNTTGGATTNNAA
>No8 Oral
CGTGAGTCTTTGTGCGTGGTGATTGAAATATCCAAGCATTCTAATATCAAGTATGAACTGG
ATAAAGAAAGCGGGGCTTTAATGGTGGATAGGGTGCTTTATGGGGCGCAAAATTACCCCG
CAAATTATGGCTTTGTGCCTAACACTTTAGGATCTGATGGCGACCCTGTAGATGCGCTTGT
TTTAAGCGATGTGGCTTTTCAAGCCGGGAGCGTGGTGAAAGCGCGCTTGGTTGGGGTTT
TGAACATGGAAGATGAAAGCGGAATGGATGAAAAATTACTCGCTCTGCCCATAGATAAGA
TTGATCCCACGCATTCCTATGTCAAAGACATTGATGATTTATCCAAACACACTTTAGATAA
AATCAAGCATTTTTTTGAAACTTACAAGGATTTAGAACCTAATAAATGGGTGAAAGTCAA
GGGGTT
TCNGTANGTGTCNCATCACCGTCGAGCTTCTCGCGCATCGGTGCCGCCACTTGC---
GGGCCAGGCTCGGCGGGCCCGGGATGATGACTGAGCGGGCCCGGTGGCGCAGGAGGAG

CTCCTCGATGACCACTCCCGCCACGATGGTCTTGCCCTCCTCCACCACGTCGGCCCCTCC
AGTCCCTCTGCGAGGACCCCCTGGAGCCNGAGCTGCGCGTCCGCTGTGAGCTTGATGTC
GGGTGCTCTGTGATCCCAAACCGACTGCTGCCCCATACCATCTCCTCAGATGACTTCGCC
CACCCCCACACCCTGGACGACTTCGCCCATGCCGTGCGCTGCGGCGACGTCACCTCAGG
AGATGGCCTCGGCTTCGCCCCGGCCTTGCACTCGGGAGTTACCCTGGAGCCCTACCAGCT
NACGCCGATGTCCT-
CGGCTCTGCCGGCCTCGCCCAGGGACTGGATGGTGACCAACTACTCGAGGATGGGGCGG
ACCATCTCGGCCGACCTGGTCATCAAACACTTCCTCTTGACCTCCCGGACCCGCTCGATG
GTNNTCCNGAGCTGGCCGANCNGGGCCNTNTCGTGGCGCGATGAGATGCGCGATGNTGT
GACNCTGNNNTTCNNCATCATGTACTCCGCGCGCCTGGNCACCGATNTCNCANCCTTACG
AATAA

>No9 Gastric

TGAGTCTTTGTGCGTGGTGATTGAAATATCCAAGCATTCTAATATCAAGTATGAACTGGAT
AAAGAAAGCGGGGCTTTAATGGTGGATAGGGTGCTTTATGGGGCACAAAATTACCCCGC
CAATTATGGCTTTGTGCCTAACACTTTAGGATCTGATGGCGACCCTGTAGATGCGCTTGTT
TTAAGCGATGTGGCTTTTCAAGCCGGGAGTGTGGTGAAAGCGCGCTTGGTTGGGGTTTT
GAACATGGAAGATGAAAGCGGAATGGATGAAAAATTACTCGCTCTACCCATAGATAAGAT
CGATCCCACGCATTCCTATGTCAAAGATATTGATGATTTATCCAAACACACTTTAGATAAA
ATCAAGCATTTTTTTGAAACTTACAAGGGATTTAGAGCCTAATAAATGGGTGAAAGTCAA
GGGGTTTGAA
TTATTTNNNNNNNGCGTTTGTTGTAAAAATTTTGTTTTGGAAGGATAAGGCAATGCTAGG
ACTTGTATTGTTATATGTTGGGATTGTTTTGATCAGCAACGGGATTTGCGGATTAACCAAA
GTCGATCCTAAAAGCACTGCGGTGATGAACTTTTTTGTGGGTGGGCTTTCCATTATTTGTA
ATGTGGTTGTCATCACTTATTCTGCACTCAACCCTACAGCCCCTGTAGAAGGTGCAGAAG
ATATTGCTCAAGTATCGCACCATTTGACTAATTTCTATGGGCCAGCAACTGGGTTATTGTTC
GGTTTTACCTACTTGTATGCGGCTATCAACCACACTTTTGGTTTGGATTGGAGGCCCTACT
CTTGGTATAGCTTATTCGTAGCGATCAATACTGTTCCTGCTGCGATTTTATCCCACTATAGC
GATATGCTTGATGATCACAAAGTGTTAGGCATCACTGAAGGCGATTGGTGGGCGATCATT
TGGTTGGCTTGGGGTGTTTTGTGGCTTACCGCTTTCATTGAAAACATCTTGAAAATCCCTT
TAGGGAAATTCACTCCATGGCTTGCTATCATTGAGGGTATTTTAACCGCTTGGATTCCTGC
TTGGTTACTCTTTATCCAACACTGGGTGTGAGATGATCATAGAGCGTTTAACAGGCAATCT
AAGG

>No9 Oral

AGTCTTTGTGTGTGGTGATTGAAATATCCAAGCATTCTAATATCAAGTATGAATTGGATAA
AGAAAGCGGGGCTTTAATGGTGGATAGGGTGCTTTATGGGGCGCAAAATTACCCCGCAA
ATTATGGCTTTGTGCCTAACACTTTAGGATCTGATGGCGACCCTGTAGATGTGCTTGTTTT

AAGCGATGTGGCTTTTCAAGCCGGGAGCGTGGTGAAAGCGCGCTTGGTTGGGGTTTTGA
ACATGGAAGATGAAAGCGGAATGGATGAAAAATTACTCGCTCTACCCATAGATAAGATCG
ATCCCACGCATTCCTATGTCAAAGACATTGATGATTTATCCAAACACACTTTAGATAAAAT
CAAGCATTTTTTTGAAACTTACAAGGATTTAGAGCCTAATAAATGGGTGAAAGTCAAGGG
GTTT
TTtgACCCGNNCAGCTGCCGTATTGACAGGATCGCGGTC------
ATGATATCCCCcCTGTCACCCTGAGNCCCGCCTNGCTGATTCTCCACCTGGATGTCATCCT
ACCTGACACGGAGGTCCTTGATCACCAAGTCCTCCGCTCCCGTGGCTGAGGGTGCCCTT
AGCTTCTTGGATAGCCCAAGTATCTGCTCCTCGTCCGACAAACT-
TCTGTTAACATTCTACCCGATTTCCCTGCACTTCCTGGTTTAAGATTGAACCTCCTGTATGC
CGAACACACTGACTCCCCTTGACTGCCGgGTAATTTTGCTTTTG---
ATAACTTTACCTCTGCCGGATCAAAGGTCCAGTCATCGAAATTACTTGTATCACCCATGTG
CTGTGAATTCTGTGTCTTCGCCATAACAACGATTCCCTCCTGAATTTGGCTTTCGCCGGAA
-
CCTGGGCCAATGTCTTGTGATAACGGTCCGACAANTTAACTATACTTGGGTTATCCTATAA
ATCCCNGTGCCCCGCACCCACTGGAATTGANGANNTGNGNATCATTCNNNNANNGNNNA
NTNNNGTATGACATCCNNNNNNNNNTAATGGNTGAGGGCNNTCTCGATCTGACNTGANGG
ATATCCTGACTACGACTCNCTGNNTGCNTGACTGTACACNGCTCACNATTGATNTGTGCT
CNGACNNNACGGCGCGCACCTANNNAATAACC
>No10 Oral
ATGCGGTTGTGGGGCGTGTGTTGAACGCTTTGGGTGAGCCTATTGATGGTAAGGGTGAG
ATAGAAACAAATGAATTTAGCCTCATCGAGCAAAAAGCCCCGGGCATTATGGACAGAAA
ATCGGTGCATGAGCCTTTGCAAACAGGCATTAAAGCCATTGATGCGTTGGTGCCTATTGG
GCGCGGGCAAAGGGAATTGATCATTGGGGATAAACAAACCGGTAAAACCACTGTAGCGA
TCGATGCGATCATTAACCAAAAAGGGCAAAATGTGATTTGTATCTATGTGGCTATTGGGCA
AAAAGAATCCACTGTCGCGCAAGTGGTCCGCAAATTAGAAGAATACGGAGCGATGGAAT
ACAGCGTCGTGATCAACGCTTCGGCTTCAGATCCAGCTGCGATGCAATACTTAGCCCCTT
ATTCAGGTGTGGCTATGGGGGAATACTTTAGAGATCATGCCCGCCATGCCCTAATCATTTA
TGATGATTTGAGTAAGCATGCTGTCGCTTACAGAGAAATTTCTTTGATTTTGAGAAGACC
CCCAGGTAGGGAGGCTTTTCCTGGAGATGTGTTTTATATCCACTCACGGCTTTTAGAAAG
AGCGGCTAAACTTTGCGATGAAAAGGGTGCAGGCTCTTTGACCGCACTCCCTATTGTGG
AAACTCAAGCGGGCGATGTTTCAGCTTATATCCCTACGAATATCATTTCTATACAGACGGG
CAAATTTTCTTAGAAACGGA
AAGTGAAACTACAAGTAAAATGAATACAAGCCATAAAACTTTAAAAACCATTGCGATTTT
AGGCCAGCNTAATGTGGGGAAAAGCTCGTTATTTAACCGCTTGGCTAGAGAAAGGATCG
CTATCACTTCAGATTTTGCAGGCACTACACGAGACATTAACAAACGAAAAATCGCATTGA

ATGGCCATGAAGTGGAATTGCTAGATACAGGGGGCATGGCTAAAGACGCTCTTTTGTCTA
AAGAAATCAAAGCCCTTAATTTAAAAGCCGCTCAAATGAGCGATTTGATTTTATATGTTGT
GGATGGCAAGTCTATCCCTAGCGATGAAGATCTTAAGCTTTTTAGAGAGGTTTTTAAGATC
AACCCTAACTGCTTTTTAGTGATCAATAAAATTGACAACGACAAAGAAAAAGAGCGAGC
TTATGCATTTTCTTCTTTTGGCATGCCAAAGAGTTTTAATATTTCTGTTTCGCACAATAGAG
GCATTAGTGCCTTAATTGATGCGATATTGAACGCGCTAAATTTAAATCAAATCATAGAGCA
GGATTTGGATGCGGATATTTTAGAAAGCCTAGAAAATAACGCATCAAAAG
>No10 Gastric
TGTTCCTGTTGGGCGATGCGGTGGACTAGCGTTAAACGCACGAAGAGTTTGATGAAAGT
TCCTGTTGGCGATGCGGTTGTGGGGCGTGTGTTGAACGCTTTGGGTGAGCCTATTGATGG
CAAGGGTGAGATAGAAACGAATGAATTTAGCCTTATTGAGCAAAAAGCCCCGGGCATTAT
GGACAGAAAATCGGTGCATGAGCCTTTGCAAACAGGTATTAAAGCCATTGATGCGTTGGT
GCCTATTGGGTGCGGGCAAAGGGAATTGATCATTGGGGATAAACAAACCGGTAAAACCA
CCGTAGCGATCGATGCGATCATTAACCAAAAAGGGCAAAATGTGATCTGTATCTATGTGG
CTATTGGGCAAAAAGAATCCACTGTCGCGCAAGTGGTCCGCAAATTAGAAGAATACGGA
GCGATGGAATACAGCGTCGTGATCAATGCTTCGGCTTCAGATTCAGCTGCGATGCAATATT
TAGCCCCTTATTCAGGTGTGGCTATGGGGGAATACTTTAGAGATCATGCCCGCCATGCCCT
AATCATTTATGATGATTTGAGTAAGCATGCTGTCGCTTACAGAGAGATTTCTTTGATTTTGA
GAAGACCCCCAGGTAGGGAGGCTTTTCCTGGAGATGTGTTTTATATCCACTCACGGCTTT
TAGAAAGAGCGGCTAAGCTTTGCGATGAAAAAGGTGCAGGCTCTTTGACCGCGCTCCCT
ATTGTGGAAACTCAAGCGGGCGATGTTTCAGCCTATATCCCTACAAATATCATTT
GTTTNTTTNNNNANNNGCGTTTGTTGTAAAAATTTTGTTTTGGAAGGATAAGGCAATGCT
AGGACTTGTATTGTTATATGTTGGGATTGTTTTAATCAGCAACGGGATTTGCGGATTAACC
AAAGTCGATCCTAAAAGCACTGCGGTGATGAACTTTTTTGTGGGTGGGCTCTCCATTATT
TGTAATGTGGTTGTCATCACTTATTCTGCGCTCAACCCTACAGCCCCTGTAGAAGGCGCA
GAGGATATTGCTCAAGTATCGCACCATTTGACTAATTTCTATGGGCCAGCAACTGGGTTAT
TGTTCGGTTTTACCTACTTGTATGCGGCTATCAACCACACTTTTGGTTTGGATTGGAGGCC
CTACTCTTGGTATAGCTTATTCGTAGCGATCAACACTGTTCCTGCTGCGATTTTATCCCACT
ATAGCGATATGCTTGATGACCACAAAGTGTTAGGCATCACTGAAGGCGATTGGTGGGCGA
TCATTTGGTTGGCTTGGGGTGTTTTGTGGCTTACCGCTTTCATTGAAAATATCTTGAAAAT
CCCTTTAGGGAAATTCACTCCATGGCTTGCTATCATTGAGGGTATTTTAACCGCTTGGATC
CCTGCTTGGTTACTCTTTATCCAACACTGGGTGTGAGATGATCATAGAGCGTTTAATAGGC
AATCTAGG
TAAAATGAATACAAGCCATAAAACTTTAAAAACCATTGCGATTTTAGGCCAGCCTAATGT
GGGGAAAAGCTCGTTATTTAACCGCTTGGCTAGAGAAAGAATCGCTATCACTTCAGATTT
TGCAGGCACTACACGAGACATTAACAAACGAAAAATCGCATTGAATGGCCATGAAGTGG

AATTGCTAGATACAGGGGGCATGGCTAAAGACGCTCTTTTGTCTAAAGAAATCAAAGCCC
TTAATTTAAAAGCCGCTCAAATGAGCGATTTGATTTTATATGTTGTGGATGGCAAGTCTATC
CCTAGCGATGAAGATCTTAAGCTTTTTAGAGAAGTTTTTAAAACCAACCCTAACTGCTTTT
TAGTGATCAATAAAATTGATAACGACAAAGAAAAAGAGCGAGCTTATGCGTTTTCTTCTT
TTGGCATGCCAAAGAGTTTTAATATTTCCGTTTCGCACAATAGAGGCATTAGTGCCTTAAT
TGATGCGATATTGAGCACGCTAAATTTAAATCAAATCATAGAGCAGGATTTGGATGCGGAT
ATTTTAGAAAGCCTAGAAAATAACGCATCAG
>No11 Gastric
TATATCAGNNAAGTGATGAGCCAACAAACCCAAATTAACACGGTAGTTGAGCGTTTTTAT
TCCCCTTTTTTAAAAGCTTTCCCCACTTTAAAAGACTTAGCGAACGCTCAATTAGAGGAG
GTTTTATTGCTCTGGAGAGGGCTTGGCTATTATTCAAGGGCTAAAAATTTAAAAAAAAGC
GCNGAAATTTGCATTAAAGAACACAACTCACAATTACCCAATGACTATCAAAGCCTGTAG
AAACTCCCCGGGATTGGCGCATACACGGCTAATGCGATTTTATGTTTTGGCTTTAGAGAA
AAGAGTGCATGCGTGGATGCCAACATCAAACGAGTGCTTTTAAGGCTTTTTGGTTTGGAT
CCTAATATCACGGCTAAAGATCTACAAATTAAAGCGAATGACTTTCTCAATCTTAATGAAA
GCTTTAATCATAACCAAGCTCTAATTGATCTAGGGGCTTTAATCTGCTCCCCTAAACCCAA
ATGCGCACTTTGCCCTTTCAATCCTTATTGTTTAGGTAA
GCATTCTAATATTAAATATGAATTGGATAAAGAAAGTGGGGCTTTAATGGTGGATAGGGTG
CTTTATGGGGCGCAAAATTACCCCGCCAATTATGGCTTTGTGCCTAACACTCTAGGATCTG
ATGGCGACCCTGTAGATGCGCTTGTTTTAAGCGATGTGGCTTTTCAAGCCGGGAGCGTGG
TGAAAGCGCGCTTGGTTGGGGTTTTGAACATGGAAGATGAAAGCGGAATGGATGAAAAA
TTACTCGCTCTACCCATAGATAAGATCGATCCCACGCATTCCTATGTCAAAGATATTGATGA
TTTATCCAAACACACTTTAGATAAAATCAAGCATTTTTTTGAAACTTATAAGGATTTAGAG
CCTAATAAATGGGTGAAAGTCAAGGGGTTTGAAAACAAAGAGA
TGAAATGAATACAAGCCATAAAACTTTAAAAACCATTGCGATTTTAGGCCAACCTAATGT
GGGGAAAAGCTCGTTATTTAACCGCCTGGCTAGAGAAAGGATCGCTATCACTTCAGATTT
TGCAGGCACTACACGAGATATTAACAAACGAAAAATCGCATTGAATGGCCATGAAGTGG
AATTGCTAGATACAGGGGGCATGGCTAAAGACGCTCTTTTGTCTAAAGAAATCAAAGCCC
TTAATTTAAAAGCCGCTCAAATGAGCGATTTGATTTTATATGTTGTAGATGGCAAGTCTATA
CCTAGCGATGAAGATCTTAAGCTTTTCAGAGAGGTTTTTAAAACCAATCCTAACTGCTTTT
TAGTGATCAATAAAATTGATAACGACAAAGAAAAAGAGCGAGCTTATGCGTTTTCTTCTT
TTGGCATGCCAAAGAGTTTTAATATTTCTGTTTCGCACAATAGAGGCATTAGCGCCTTAAT
TGATGCGATATTGAACGCGCTAAATTTAAATCAAATCATAGAGCAGGATTTGGATGCAGAT
ATTTTAGAAAGCCTAGAAAATAA
>No11 Oral
GGGGGGGGGGATGGAAATTTCCAACCAGCATTCTAATATCAAGTATGAACTGGATAAAGA

AAGCGGGGCTTTAATGGTGGATAGGGTGCTTTATGGGGCGCAAAATTACCCCGCAAATTA
TGGCTTTGTGCCTAACACTCTAGGATCTGATGGCGACCCTGTAGATGTGCTTGTTTTAAGC
GATGTGGCTTTTCAAGCCGGGAGCGTGGTGAAAGCGCGCTTGGTTGGGGTTTTGAACAT
GGAAGATGAAAGCGGAATGGATGAAAAATTACTCGCTCTACCCATAGATAAGATCGATCC
CACGCATTCCTATGTCAAAGACATTGATGATTTATCCAAACACACTTTAGATAAAATCAAG
CATTTTTTGAAACTTACAAGGATTTAGAGCCAATAAATGGG
TACAAGTAAAATGAATACAAGCCATAAAACTTTAAAAACCATTGCGATTTTAGGCCAGCC
TAATGTGGGGAAAAGCTCGTTATTTAACCGCTTGGCTAGAGAAAGGATCGCTATCACTTC
AGATTTTGCAGGCACTACACGAGACATTAACAAACGAAAAATCGCATTGAATGGCCATG
AAGTGGAATTGCTAGATACAGGGGGCATGGCTAAAGACGCTCTTTTGTCTAAAGAAATCA
AAGCCCTTAATTTAAAAGCCGCTCAAATGAGCGATTTGATTTTATATGTTGTGGATGGCAA
GTCTATCCCTAGCGATGAAGATCTTAAGCTTTTTAGAGAGGTTTTTAAGATCAACCCTAAC
TGCTTTTTAGTGATCAATAAAATTGATAACGACAAAGAAAAAGAGCGAGCTTATGCGTTT
TCTTCTTTTGGCATGCCAAAGAGTTTTAACATTTCCGTTTCGCACAATAGAGGCATTAGCG
CCTTAATTGATGCGATATTGAGCGCGCTAAATTTAAATCAAATCATAGAGCAGGATTTGGA
TGCGGATATTTTAGAAAGCCTAGAAAATAACGCATCAAAAGA
>No12 Oral
TTtAtCtGTttTTTATTCCCCTTTTTTAGAAGCTTTCCCCACTTTAAAAGATTTAGCGAACGCT
CAATTAGAAGAGGTTTTATTGCTCTGGAGAGGGCTTGGCTACTATTCAAGGGCTAAttAAA
TTTAAAAAAAAGCGCTGAAATTccTGCGTTAAAGAGCATGACTCACAATTACCCAcATGAC
TATCAAAGCCTGTTGAAACTCCCCGGGATTGGCGCATACACGGCTAATGCGATTTTATGTT
TTGtGCTTTAGAGAAAAGAGAGCATGCGTAGATGCTAACATCAAACGGGTGCTTTTAAGG
CTTTTTGGTTTGGATCCTAATATCACGGCTAAAGACTTACAAATTAAAGCGAATGACTTTC
TCAATCTTAAT--
GAAAGCTTTAATCATAACCAAGCCCTAATTGATCTTGGACTAGGGGCTTTAATCTGCTCCC
CTAAACCCAAATGCGCACTTTGCCCTTTCAATCCTTATTGCTTAAGTAcA
AGTCTTTGTGTGTGGTGATTGAAATATCCAAGCATTCTAATATCAAGTATGAACTGGATAA
AGAAAGCGGGGCTTTAATGGTGGATAGGGTGCTTTATGGGGCGCAAAATTACCCCGCAA
ATTATGGCTTTGTGCCTAACACTCTAGGATCTGATGGCGACCCTGTAGATGTGCTTGTTTT
AAGCGATGTGGCTTTTCAAGCCGGGAGCGTGGTGAAAGCGCGCTTGGTTGGGGTTTTGA
ACATGGAAGATGAAAGCGGAATGGATGAAAAATTACTCGCTCTACCCATAGATAAGATCG
ATCCCACGCATTCCTATGTCAAAGACATTGATGATTTATCCAAACACACTTTAGATAAAAT
CAAGCATTTTTTTGAAACTTACAAGGATTTAGAGCCTAATAAATGGGTGAAAGTCAAGGG
GTTTGAAAAACAAAGAGA
GTAAAATGAATACAAGCCATAAAACTTTAAAAACCATTGCGATTTTAGGCCAGCCTAATG
TGGGGAAAAGCTCGTTATTTAACCGCCTGGCTAGAGAAAGGATCGCTATCACTTCAGATT

TTGCAGGCACTACACGAGACATTAACAAACGAAAAATCGCATTGAATGGCCATGAAGTG
GAATTGCTAGATACAGGGGGCATGGCTAAAGACGCTCTTTTGTCTAAAGAAATCAAAGCC
CTTAATTTAAAAGCCGCTCAAATGAGCGATTTGATTTTATATGTTGTGGATGGTAAGTCTAT
CCCTAGCGATGAAGATCTTAAGCTTTTTAGAGAGGTTTTTAAGATCAACCCTAACTGCTTT
TTAGTGATCAATAAAATTGACAACGACAAAGAAAAAGAGCGAGCTTATGCATTTTCTTCT
TTTGGCATGCCAAAGAGTTTTAATATTTCTGTTTCGCACAATAGAGGCATTAGTGCCCTAA
TTGATGCGATATTGAACGCGCTAAATTTAAATCAAATCATAGAGCAGGATTTGGATGCGGA
TATTTTAGAAAGCCTAGAAAATAACGCATC
>No12 Gastric
TTTTTTTATATCAGCGAAGTGATGAGCCAACAAACCCAAATCAACACGGTAGTTGAGCGT
TTTTATTCCCCTTTTTTAAAAGCTTTCCCCACTTTAAAAGACTTAGCGAGCGCTCCATTAG
AGGAGGTTTTATTGCTCTGGAGAGGGCTTGGTTATTATTCAAGGGCTAAAAATTTAAAAA
AAAGCGCTGAAATTTGCGTTAAAGAACATGACTCACAATTACCCAATGACTATCAAAGCC
TGTTGAAACTCCCCGGGATTGGTGCATACACGGCTAATGCGATTTTATGTTTTGGCTTTAG
AGAAAAGAGCGCATGCGTAGATGCTAACATCAAACGGGTGCTTTTAAGGCTTTTTGGTCT
GGATCCAAATATCACGGCTAAAGACTTACAAATTAAGGCGAATGACTTTCTTAATCTTAAT
GAAAGCTTTAATCATAACCAAGCCCTAATTGATCTAGGGGCTTTAATCTGCTCCCCTAAAC
CCAAATGCGCACTTTGCCCTTTCAATCCTTATTGGTTTAGGTAA
AGCCATGACGCTGAGTCTTTGTGCGTGGTGATTGAAATATCCAAGCATTCTAATATCAAGT
ATGAATTGGATAAAGAAAGCGGGGCTTTAATGGTGGATAGGGTGCTTTATGGGGCGCAAA
ATTACCCCGCCAATTATGGCTTTGTGCCTAACACTTTAGGATCTGATGGCGACCCTGTAGA
TGCGCTTGTTTTAAGCGATGTGGCTTTTCAAGCCGGGAGCGTGGTGAAAGCGCGCTTGG
TTGGGGTTTTGAACATGGAAGATGAAAGCGGAATGGATGAAAAATTACTCGCTCTACCCA
TAGATAAGATCGATCCCACGCATTCCTATGTCAAAGATATTGATGATTTATCCAAACACACT
TTAGATAAAATCAAGCATTTTTTTGAAACTTACAAGGATTTAGAACCTAATAAATGGGTGA
AAGTCAAGGGGTTT
TACAAGTGAAATGAATACAAGCCACAAAACTTTAAAAACCATTGCGATTTTAGGCCAGCC
TAATGTGGGGAAAAGCTCGTTATTTAACCGCCTAGCTAGAGAAAGGATCGCTATCACTTC
AGATTTTGCAGGCACTACACGAGATATTAACAAACGAAAAATCGCATTGAATGGCCATGA
AGTGGAATTGCTGGATACAGGGGGCATGGCTAAAGACGCTCTTTTGTCTAAAGAAATCAA
AGCCCTTAATTTAAAAGCCGCTCAAATGAGCGATTTGATTTTATATGTTGTGGATGGCAAG
TCTATCCCTAGCGATGAAGATCTTAAGCTTTTTAGAGAGGTTTTTAAAACCAACCCTAACT
GCTTTTTAGTGATCAATAAAATTGACAACGACAAAGAAAAAGAGCGAGCTTATGCGTTTT
CTTCTTTTGGCATGCCAAAGAGTTTTAACATTTCCGTTTCGCACAATAGAGGCATTAGCGC
CTTAATTGATGCGATATTGAGCGCGCTAAATTTAAATCAAATCATAGAGCAGGATTTGGAT
GCAGATATTTTAGAAAGCCTAGAAAATAACGCACCAAAA

>No13 Gastric
ACGCACGAAGAGTTTGATGAATGTTCCTGTTGGGGATGCGGTTGTGGGGCGTGTGTTGA
ACGCTTTGGGTGAGCCTATTGATGGCAAGGGTGAGATAGAAACGAATGAGTTTAGCCTCA
TCGAGCAAAAAGCCCCGGGCATTATGGACAGAAAATCGGTGCATGAGCCTTTGCAAACA
GGTATTAAAGCCATTGATGCGTTGGTGCCTATTGGGCGCGGGCAAAGGGAATTGATCATT
GGGGATAAACAAACCGGTAAAACCACCGTAGCGATCGATGCGATCATTAACCAAAAAGG
GCAAAATGTGATCTGTATCTATGTGGCTATTGGGCAAAAAGAATCCACTGTCGCGCAAGT
GGTCCGCAAATTAGAAGAATACGGAGCGATGGAATACAGCGTCGTGATCAACGCTTCGG
CTTCAGATTCGGCTGCGATGCAATATTTAGCCCCTTATTCAGGTGTGGCTATGGGGGAATA
CTTTAGAGATCATGCCCGCCATGCCCTAATCATTTATGATGATTTGAGTAAGCATGCTGTCG
CTTACAGAGAAATTTCTTTGATTTTGAGAAGACCCCCAGGTAGGGAGGCTTTTCCTGGAG
ATGTGTTTTATATCCACTCACGGCTTTTAGAAAGAGCGGCTAAACTTTGCGATGAAAAGG
GTGCAGGCTCTTTGACCGCACTCCCTATTGTGGAAACTCAAGCGGGCGATGTTTCAGCCT
ATATCCCTACGAATATCATTTCTATTACAGACGGACAAATTTTCTTA
GGAGCAGATTGANGCCCCTATGATCTATTAGTGGGTTGNGTTATGATTAAAGCTGG-
GTTAANATTGAGAAAGTCATTCNCCTTAATTTGTAAGTCTTTAGCCGTGATATTTGGATCC
AAACCAAAAAGCCTTAAAANGACTCGNTTGATGTTTGCATCCACGCATGCCCTCTTTTCC
CTAAAGCCAAAACNTAAAATCGCNTTACCCGTGTCTGCACCAATCCCGGGGAGTTTCAA
CAGGCTTTGATAGTCATTGGGTAATTGTGAGTTGTGTTCTTTAACGCAAATTTCAGCGCTT
TTTTTTAAATTTTTAGCCCTTGAATAATAACCAAGCCCCCTCCAGAGCAATAAAACCTCTT
CTAATTGAGCGTTCGATAAGTCTTTTAAAGTGGGGAAAGCTTCTAAAAAAGGGGAATAAA
AACGCTCAAATTAACCCGTTTTATTTGTTTTT
>No13 Oral
GATGCGGTTGTGGGGCGTGTGTTGAACGCTTTGGGTGAGCCTATTGATGGCAAGGGTGA
GATAGAAACGAATGAATTTAGCCTTATTGAGCAAAAAGCCCCGGGCATTATGGACAGAAA
ATCGGTGCATGAGCCTTTGCAAACAGGTATTAAAGCCATTGATGCGTTGGTGCCTATTGG
GCGTGGGCAAAGGGAATTGATCATTGGGGACAAACAAACCGGTAAAACCACCGTAGCG
ATCGATGCGATCATTAACCAAAAAGGGCAAAATGTGATCTGTATCTATGTGGCTATTGGGC
AAAAAGAATCCACTGTCGCGCAAGTGGTGCGCAAATTAGAAGAATACGGAGCGATGGAA
TACAGCGTCGTGATCAACGCTTCGGCTTCAGATTCAGCTGCGATGCAATATTTAGCCCCTT
ATTCAGGTGTGGCTATGGGAGAATACTTTAGAGATCATGCCCGCCATGCCTTAATCATTTAT
GATGATTTGAGTAAGCATGCTGTCGCTTACAGAGAAATTTCTTTGATTTTGAGAAGACCC
CCAGGTAGGGAGGCTTTTCCTGGAGATGTGTTTTATATCCACTCACGGCTTTTAGAAAGA
GCAGCTAAACTTTGCGATGAAAAGGGTGCAGGCTCTTTGACCGCGCTCCCTATTGTGGA
AACTCAAGCGGGCGATGTTTCAGCCTATATCCCTACGAATATCATTTCTATTACAGACGGG
CAAATTTTCTTAGAAACGGATGT

TATTATTCAAGGGCTAAAAATTTAAAAAAAAGCGCTGAAATTTGCGT---TAAAGAAC-
ATGACTCACAATTACCCA-ATG--AC--TATCAAAGCCTGTTGAAACTCCCCGGGA-
TTGGCGCATACACGGCTAA-
TGCGATTTTATGTTTTGGCTTTAGAGAAAAGAGAGCATGCGTGGATGCTAACATCAAACG
GGTGCctgTTTTAAGGCTTTTTGGTTTGGATCCTAATAcTCACGGCTAAAGACTgTACAAATT
AAAGCGAATcccGACTTTCATTTCAATCTTAATGAAAGCTTTAATCAT-
AACCAAGCCCTAATTGATCTAGGGGCTTTAATCTGCTCCCCTAAACCCAAATGCGCAATTT
GCCCTTTCAATCCTTATGGGTTTAGGTAA
>No15 Gastric
GCTTGAAAATTGAATTGGGCGGTGTGCCTTATAGGATCGTAGAATACCAGCATGTCAAGC
CCGGCAAGGGTGCGGCTTTTGTGCGCGCGAAAATCAAGTCGTTTTTAGATGGTAAGGTG
ATTGAAAAGACTTTCCATGCGGGGGATAAGTGCGAAGAGCCTAATCTGGTTGAAAAAAC
GATGCAATACCTTTATCACGATGGCGATACATACCAATTCATGGATATAAAGAACTATGAG
CAAATCGCTTTGAACGACTCTCAAGTGGGCGAGGCTTCTAAATGGATGCTAGACGGCATG
CAAGTGCAGGTTTTATTGCATAATGACAAGGCGATTTCAGTGGATGTGCCGCAAGTTGTG
GCTTTAAAGATTGTAGAAACAGCCCCTAATTTTAAGGGCGATACTTCAAGTGCGAGCAAA
AAACCAGCGACTTTAGAAACCGGTGCGGTCGTGCAAGTGCCTTTCCATGTTTTAGAGGG
TGAGATCATTAAGNTCAATACAGAAACAGAAGAGTATCTTGAAAAGGTGAAGAATCATTA
AAGTCAATACA
TTTATATCAGTGAAGTGATGAGCCAACAAACCCAAATCAACACGGTAATTGAGCGTTTTT
ATTCCCCTTTTTTAAAAGCTTTCCCCACTTTAAAAGATTTAGCGAACGCTCAATTAGAAGA
GGTTTTATTGCTCTGGAGAGGGCTTGGCTATTATTCAAGGGCTAAAAATTTAAAAAAAAG
CGCTGAAATTTGCGTTAAAGAGCATAACTCACAATTACCCAATGACTATCAAAGCCTGTT
GAAACTCCCCGGGATTGGCGCATACACGGCTAATGCGATTTTATGTTTTGGCTTTAGAGA
AAAGAGCGCATGCGTGGATGCTAACATCAAACGGGTGCTTTTAAGGCTTTTTGGTCTGGA
TCCAAATATCACGGCTAAAGACTTACAAATTAAGGCGAATGACTTTCTCAATCTTAATGAA
AGCTTTAATCATAACCAAGCCCTAATTGATCTAGGGGCTTTAATCTGCTCCCCTAAACCCA
AATGCGCACTTTGCCCTTTCAATCCTTATTGTTTAGGTAA
TACAACCGTGATCATTCCAGCCATTGTTGGGGGTATCGCTACAGGTGCTGCTGTAGGAAC
GGTCTCAGGGCTTCTTGGCTGGGGGCTCAAACAAGCCGAAGAAGCGAATAAAACCCCG
GATAAACCCGATAAAGTTTGGCGCATTCAAGCAGGAAGAGGTTTTAATGAATTCCCTAAC
AAGGAATACGACTTATACAAATCCCTTTTATCCAGTAAGATTGATGGAGGTTGGGATTGGG
GGAATGCCGCTAGGCATTATTGGGTCAAAGGCGGGCAATGGAACAAGCTTGAAGTGGAT
ATGAAAGATGCTGTAGGGACTTATAAGCTTTCAGGGCTAAGAAACTACACTGGTGGGGAT
TTAGATGTCAATATGCAAAAAGCCACTTTGCGCTTGGGCCAATTCAATGGCAATTCTTTCA
CAAGCTTTAAGGATAGTGCTGATCGCACCACGAGAGTGGATTTCAACGCTAAAAATATCT

CAATTGATAATTTTTTAGAAATCAATAATCGTGTGGGTTCTGGAGCCGGGAGAAAAGCTA
GCTCTACGGTTTTAACTTTGCAAGCTTCAGAGGGGATCACTAGCGGTAAAAACGCTGAA
ATTTCTCTTTATGATGGCGCCACGCA
>No15 0ral
ATTGAATTGGGCGGTGTGCCTTATAGGATCGTAGAATACCAACATGTCAAGCCCGGCAAG
GGTGCGGCTTTTGTGCGCACGAAAATCAAGTCGTTTTTAGATGGTAAGGTGATTGAAAAG
ACTTTCCATGCGGGGGATAAGTGCGAAGAGCCTAATCTGGTTGAAAAAACGATGCAATAC
CTTTATCACGATGGCGATACATACCAATTCATGGATATAGAGAGCTATGAGCAAGTCGCCT
TGAACGACTCTCAAGTGGGCGAGGCTTCTAAATGGATGCTAGACGGCATGCAAGTGCAG
GTTTTATTGCATAATGACAAGGCGATTTCAGTGGATGTGCCGCAAGTTGTGGCTTTAAAG
ATTGTAGAAACAGCCCCTAATTTTAAGGGCGATACTTCAAGCGCGAGCAAAAAACCAGC
GACTTTAGAAACCGGTGCGGTTGTGCAAGTGCCTTTCCATGTTTTAGAGGGTGAGATCAT
TAAAG
TTTTATATCAGTGAAGTGATGAGCCAACAAACCCAAATCAACACGGTAATTGAGCGTTTT
TATTCCCCTTTTTTAGAAGCTTTCCCCACTTTAAAAGATTTAGCGAACGCTCAATTAGAAG
AGGTTTTATTGCTCTGGAGAGGGCTTGGCTACTATTCAAGGGCTAAAAATTAAAAAAAA
GCGCTGAAATTTGCGTTAAAGAGCATGACTCACAATTACCCAATGACTATCAAAGCCTGT
TGAAACTCCCCGGGATTGGCGCATACACGGCTAATGCGATTTTATGTTTTGGCTTTAGAGA
AAAGAGAGCATGCGTAGATGCTAACATCAAACGGGTGCTTTTAAGGCTTTTTGGTTTGGA
TCCTAATATCACGGCTAAAGACTTACAAATTAAAGCGAATGACTTTCTCAATNNNNANGA
AAGCTTTANTCATAACCAAGCCCTAATTGATCTAGGGGCTTTAATCTGCTCCCCTAAACCC
AAATGCGCACTTTGCCCTTTCAATCCTTATTGTTTAGGTAA
TCTGCTGTAGGGAACGGTCTCAGGGCTTCTTAGCTGGGGGCTCAAACAAGCCGAAGAAG
CCAATAAAACCCCAGATAAACCCGATAAAGTTTGGCGCATTCAAGCAGGAAGAGGCTTC
AATAATTTTCCTCACAAGGAATACGACTTATACAAATCCCTTTTATCCAGTAAGATTGATG
GAGGCTGGGATTGGGGGAATGCCGCTAGGCATTATTGGGTCAAAGGCGGGCAATGGAAC
AAGCTTGAAGTGGATATGAAAGACGCTGTAGGGACTTATAAACTTTCAGGCCTTATCAAC
TTTACTGGTGGGGATTTAGATGTCAATATGCAAAAAGCCACTTTGCGCTTGGGCCAATTC
AATGGCAATTCTTTCACAAGCTATAAGGATAGTGCTGATCGCACCACGAGAGTGGATTTC
AACGCTAAAAATATCTTAATTGATAATTTTTTAGAAATCAATAATCGTGTGGGTTCTGGAG
CCGGGAGGAAAGCCAGCTCTACGGTTTTAACTTTGCAAGCTTCAGAAGGGATCACTAGC
AGTAAAAACGCTGAAATTTCTCTTTATGNGNCNCCNCGCAA
>No16 Oral
CGATGCGGTTGTGGGGCGTGTGTTGAACGCTTTGGGTGAGCCTATTGATGGCAAGGGTG
AGATAGAAACGAATGAATTTAGCCTTATTGAGCAAAAAGCCCCGGGCATTATGGACAGAA
AATCGGTGCATGAGCCTTTGCAAACAGGTATTAAAGCCATTGATGCGTTGGTGCCTATTG

GGCGCGGGCAAAGGGAATTGATCATTGGGGATAAACAAACCGGTAAAACCACCGTAGCG
ATCGATGCGATCATTAACCAAAAAGGGCAAAATGTGATCTGTATCTATGTGGCTATTGGGC
AAAAAGAATCCACTGTCGCGCAAGTGGTCCGCAAATTAGAAGAATACGGAGCGATGGAA
TACAGCGTCGTGATCAATGCTTCGGCTTCAGATTCAGCTGCGATGCAATATTTAGCCCCTT
ATTCAGGTGTGGCTATGGGGGAATACTTTAGAGATCATGCCCGCCATGCCCTAATCATTTA
TGATGATTTGAGTAAGCATGCTGTCGCTTACAGAGAGATTTCTTTGATTTTGAGAAGACC
CCCAGGTAGGGAGGCTTTTCCTGGAGATGTGTTTTATATCCACTCACGGCTTTTAGAAAG
AGCGGCTAAGCTTTGCGATGAAAAAGGTGCAGGCTCTTTGACCGCGCTCCCTATTGTGG
AAACTCAAGCGGGCGATGTTTCAGCCTATATCCCTACAAATATCATTTCTATTACAGACGG
GCAAATTTTCTTAGAAACGGATTTGTTTTATTCAGGGATCCGCCCGGCTATCAATGTGGGC
TTGTCGGGT

GTTATTNNNNAGGTGCGTTTGTTGTAAAAATTTTGTTTTGGAAGGATAAGGCAATGCTAG
GACTTGTATTGTTATATGTTGGGATTGTTTTAATCAGCAACGGGATTTGCGGATTAACCAA
AGTCGATCCTAAAAGCACTGCGGTGATGAACTTTTTTGTGGGTGGGCTCTCCATTATTTGT
AATGTGGTTGTCATCACTTATTCTGCGCTCAACCCTACAGCCCCTGTAGAAGGCGCAGAA
GATATTGCTCAAGTATCGCACCATTTGACTAATTTCTATGGGCCAGCAACTGGGTTATTGTT
CGGTTTTACCTACTTGTATGCGGCCATCAACCACACTTTTGGTTTGGATTGGAGGCCCTAC
TCTTGGTATAGCTTATTCGTAGCGATCAACACTGTTCCTGCTGCGATTTTATCCCACTATAG
CGATATGCTTGATGACCACAAAGTGTTAGGCATCACTGAAGGCGATTGGTGGGCGATCAT
TTGGTTGGCTTGGGGTGTTTTGTGGCTTACCGCTTTCATTGAAAACATCTTGAAAATCCCT
TTAGGGAAATTCACTCCATGGCTTGCTATCATTGAGGGTATTTTAACCGCTTGGATCCCTG
CTTGGTTGCTCTCTATCCAACACTGGGTGTGAGATGATCATAGAGCGTTTAATAGGCAATC
TAANGGAATTTAAACA

TTTGATTAAAGTGAAACTACAAGTAAAATGAATACAAGCCATAAAACTTTAAAAACCATT
GCGATTTTAGGCCAGCCTAATGTGGGGAAAAGCTCGTTATTTAACCGCTTGGCTAGAGAA
AGGATCGCTATCACTTCAGATTTTGCAGGCACTACACGAGACATTAACAAACGAAAAATC
GCATTGAATGGTCATGAAGTGGAATTGCTAGATACAGGGGGCATGGCTAAAGACGCTCTT
TTGTCTAAAGAAATCAAAGCCCTTAATTTAAAAGCCGCTCAAATGAGCGATTTGATTTTAT
ACGTTGTGGATGGCAAATCTATCCCTAGCGATGAAGATCTTAAGCTTTTTAGAGAGGTTTT
TAAAACTAACCCTAACTGTTTTTTAGTGATCAATAAAATTGATAACGACAAAGAAAAAGA
GCGAGCTTATGCGTTTTCTTCTTTTGGCATGCCGAAGAGTTTTAACATTTCCGTTTCGCAC
AATAGAGGCATTAGTGCCTTAATTGATGCGATATTGAGCACGCTAAATTTAAATCAAATCA
TAGAGCAAGATCTAGATGCGGATATTTTAGAAAGCCTAGAAAATAACGCACCAAAAGA

>No16 Gastric

GCGCGGCTATCGACGAGTTCGTATTCTTCGGTTCCGCGTTCGACTCAAGTTCCAGTGAGC
ACTTCACCAACGTTGCCCTCATAGATACCATTCAGGGCTCCGGCTGGGCAATTCATGATC

GCATCGATCGCTGGGACACCGTGGGCAAGCGCCTCATCATTTACAGAGCAGCTGTACGA
CCAGCACCCGGGCAACATCTCGGTTGCACTGATTCCCGTTCTGCAGCTCGATATGTGGGA
GCACCGGCCTTCTACCTGGACTACCAGAAGCGTGAAGGGCGACTACGTCAAGGCATTCT
GGACACATCGTGAACTGGGCAGACGTCGCTGAGCCCAGCTGTTCGCACGCGCAGTTTCG
CAGCCACCAAGGGTTTGGTGCTTCCCGAGTAGTCCTCAAGGATTAACGACGCGCTCGCA
CTCAGGGATCGTATCGTATACGGGCGGGTGC
GNNTTATTTNNNNNNNNTGCGTTTGTTGTAAAAATTTTGTTTTGGAAGGATAAGGCAATG
CTAGGACTTGTATTGTTATATGTTGGGATTGTTTTGATCAGCAATGGGATTTGCGGATTAAC
CAAAGTCGATCCTAAAAGCACTGCGGTGATGAACTTTTTTGTGGGTGGGCTTTCCATTAT
TTGTAATGTGGTTGTCATCACTTATTCTGCGCTCAACCCTACAGCCCCTGTAGAAGGCGCA
GAGGATATTGCTCAAGTATCGCACCATTTGACTAATTTCTATGGGCCAGCAACTGGGTTAT
TGTTCGGTTTTACCTACTTGTATGCGGCTATCAACCACACTTTTGGTTTGGATTGGAGGCC
CTACTCTTGGTATAGCTTATTCGTAGCGATCAACACTGTTCCTGCTGCGATTTTATCCCACT
ATAGCGATATGCTTGATGACCACAAAGTGTTAGGCATCACTGAAGGCGATTGGTGGGCGA
TCATTTGGTTGGCTTGGGGTGTTTTGTGGCTTACCGCTTTCATTGAAAACATCTTGAAAAT
CCCTTTAGGGAAATTCACTCCATGGCTTGCTATCATTGAGGGTATTTTAACCGCTTGGATT
CCTGCTTGGTTACTCTTTATCCAACACTGGGTGTGAGATGATCATAGAGCGTTTAATAGGC
AATCTAAGGAAANNTTAAACANNNTGNATTTCA
TTTGATTAAAGTGAAACTACAAGTAAAATGAATACAAGCCATAAAACTTTAAAAACCATT
GCGATTTTAGGCCAGCCTAATGTGGGGAAAAGCTCGTTATTTAACCGCTTAGCTAGAGAA
AGGATCGCTATCACTTCAGATTTTGCAGGCACTACACGAGACATTAACAAACGAAAAATC
GCATTGAATGGTCATGAAGTGGAATTGCTAGATACAGGGGGCATGGCTAAAGACGCTCTT
TTGTTTAAAGAAATCAAAGCCCTTAATTTAAAAGCCGCTCAAATGAGCGATTTGATTTTAT
ATGTTGTAGATGGCAAGTCTATACCTAGCGATGAAGATCTTAAGCTTTTTAGAGAGGTTTT
TAAAACCAACCCTAACTGCTTTTTAGTGATCAATAAAATTGATAACGACAAAGAAAAAGA
GCGAGCTTATGCGTTTTCTTCTTTTGGCATGCCAAAGAGTTTTAATATTTCCGTTTCGCAC
AATAGAGGCATTAGCGCCTTAATTGATGCGATATTGAACGCGCTAAATTTAAATCAAATCA
TAGAGCAGGATTTGGATGCGGATATTTTAGAAAGCCTAGAAAATAACGCATCAAA
>No17 Oral
TCTTTAAGCGCGAATTTCAGGCGTTTTTATTCCCCTTTTTTAGAAGCTTTCCCCACTTTAA
AAGATTTAGCGAACGCTCAATTAGAAGAGGTTTTATTGCTCTGGAGAGGGCTTGGCTACT
ATTCAAGGGCTAAAAATTTAAAAAAAAAGCGCTGAAATTTGCGTTAAAGAGCATGACTCA
CAATTACCCAATGACTATCAAAGCCTGTTGAAACTCCCCGGGATTGGCGCATACACGGCT
AATGCGATTTTATGTTTTGGCTTTAGAGAAAAGAGAGCATGCGTAGATGCTAACATCAAA
CGGGTGCTTTTAAGGCTTTTTGGTTTGGATCCTAATATCACGGCTAAAGACTTACAAATTA
AAGCGAATGACTTTCTCAATCTTAATGAAAGCTTTAATCATAACCAAGCCCTAATTGATCT

AGGGGCTTTAATCTGCTCCCCTAAACCCAAATGCGCACTTTGCCCTTTCAATCCTTATTGT
TTAGGTAA

TTTGATTAAAGTGAAACTACAAGTGAAATGAATACAAGCCATAAAACTTTAAAAACCATT
GCGATTTTAGGCCAGCCTAATGTGGGGAAAAGCTCGTTATTTAACCGCTTAGCTAGAGAA
AGGATCGCTATCACTTCAGATTTTGCAGGCACTACACGAGACATTAACAAACGAAAAATC
GCATTGAATGGCCATGAAGTGGAATTGCTAGATACAGGGGGCATGGCTAAAGACGCTCTT
TTGTCTAAAGAAATCAAAGCCCTTAATTTAAAAGCCGCTCAAATGAGCGATTTGATTTTAT
ATGTTGTGGATGGCAAGTCTATCCCTAGCGATGAAGATCTTAAGCTTTTTAGAGAGGTTTT
TAAGATCAACCCTAACTGCTTTTTAGTGATCAATAAAATTGATAACGACAAAGAAAAAGA
GCGAGCTTATGCGTTTTCTTCTTTTGGCATGCCAAAGAGTTTTAACATTTCCGTTTCGCAC
AATAGAGGCATTAGCGCCTTAATTGATGCGATATTGAGCGCGCTAAATTTAAATCAAATCA
TAGAGCAGGATTTGGATGCGGATATTTTAGAAAGCCTAGAAAATAACGCATCAAAAGAAG

>No17 Gastric

TATATCAGCGAAGTGATGAGCCAACAAACCCAAATCAACACGGTAGTTGAGCGTTTTTAT
TCCCCTTTTTTAAAAGCTTTCCCCACTTTAAAAGATTTAGCGAACGCTCAATTAGAAGAG
GTTTTATTGCTCTGGAGAGGGCTTGGCTACTATTCAAGGGCTAAAAATTTAAAAAAAAGC
GCTGAAATTTGCGTTAAAGAGCATGACTCACAATTACCCAATGACTATCAAAGCCTATTAA
AACTCCCCGGGATTGGCACATACACGGCTAATGCGATTTTATGTTTTGGCTTTAGAGAAA
AGAGAGCATGCGTAGATGCTAACATCAAACGGGTGCTTTTAAGGCTTTTTGGTCTGGATC
CAAATATCACGGCTAAAGACTTACAAATTAAGGCGAATGACTTTCTCAATCTTAATGAAA
GCTTTAATCATAACCAAGCCCTAATTGATCTAGGGGCTTTAATCTGCTCCCCTAAACCCAA
ATGCGCACTTTGCCCTTTCAATCCTTANNNTTTTAGGTAA

AGGTTATTTNNNNNNGTGCGTTTGTTGTAAAAATTTTGTTTTGGAAGGATAAGGCAATGC
TAGGACTTGTATTGTTATATGTTGGGATTGTTTTAATCAGCAACGGGATTTGCGGATTAACT
AAAGTCGATCCTAAAAGCACTGCGGTGATGAACTTTTTTGTGGGTGGGCTTTCCATTATTT
GTAATGTGGTTGTCATCACTTATTCTGCGCTCAACCCTACAGCCCCTGTAGAAGGCGCAG
AGGATATTGCTCAAGTATCGCACCATTTGACTAATTTCTATGGGCCAGCAACTGGGTTATT
GTTCGGTTTTACCTACTTGTATGCGGCTATCAACCACACTTTTGGTTTGGATTGGAGGCCT
TACTCTTGGTATAGCTTATTCGTAGCGATCAACACTGTTCCTGCTGCGATTTTATCCCACTA
TAGCGATATGCTTGATGACCACAAAGTGTTAGGCATCACTGAAGGCGATTGGTGGGCGAT
CATTTGGTTGGCTTGGGGTGTTTTGTGGCTTACCGCTTTCATTGAAAACATCTTGAAAATC
CCTTTAGGGAAATTCACTCCATGGCTTGCTATCATTGAGGGTATTTTAACCGCTTGGATTC
CTGCTTGGTTACTCTTTATCCAACACTGGGTGTGAGATGATCATAGAGCGTTTAATAGGCA
ATCTAAGGGAATTTAAACACCNTGGATTTCAA

TTAAAGTGAAACTACAAGTGAAATGAATACAAGCCATAAAACTTTAAAAACCATTGCGAT
TTTAGGCCAGCCTAATGTGGGGAAAAGCTCGTTATTTAACCGCTTAGCTAGAGAAAGGAT

CGCTATCACTTCAGATTTTGCAGGCACTACACGAGACATTAACAAACGAAAAATCGCATT
GAATGGCCATGAAGTGGAATTGCTAGATACAGGGGGCATGGCTAAAGACGCTCTTTTGTC
TAAAGAAATCAAAGCCCTTAATTTAAAAGCCGCTCAAATGAGCGATTTGATTTTATATGTT
GTGGATGGCAAGTCTATCCCTAGCGATGAAGATCTTAAGCTTTTTAGAGAGGTTTTTAAG
ATCAACCCTAACTGCTTTTTAGTGATCAATAAAATTGATAACGACAAAGAAAAAGAGCGA
GCTTATGCGTTTTCTTCTTTTGGCATGCCAAAGAGTTTTAACATTTCCGTTTCGCACAATA
GAGGCATTAGCGCCTTAATTGATGCGATATTGAGCGCGCTAAATTTAAATCAAATCATAGA
GCAGGATTTGGATGCGGATATTTTAGAAAGCCTAGAAAATAACGCATCAAA
>No18 Oral
ATATCAGTGAAGTGATGAGCCAACAAACCCAAATCAACACGGTAATTGAGCGTTTTTATT
CCCCTTTTTTAGAAGCTTTCCCCACTTTAAAAGACTTGGCGAACGCTCAATTAGAAGAGG
TTTTATTGCTCTGGAGAGGGCTTGGTTATTATTCAAGGGCTAAAAATTTAAAAAAAAGCG
CTGAAATTTGCGTTAAAGAACACAACTCGCAATTACCCAATGACTATCAAAGCCTGTTAA
AACTCCCCGGGATTGGCGCATACACGGCTAATGCGATTTTATGTTTTGGCTTTAGAGAAA
AGAACGCATGCGTGGATGCTAACATCAAACGAGTGCTTTTAAGGCTTTTTGGTTTGGATC
CAAATATCACAGCTAAAGATCTACAAATTAAGGCGAATGACTTTCTCAATCTTAATGAAAG
CTTTAATCATAACCAAGCCCTAAATTGATCTAGGGGCTTTAATCTGCTCCCCTAAACCCAA
ATGCACGATTTGCCCTTCCAATCCTTATTGTTTAGGTA
TGATGTTATACGCATACATATATATTCTCTCTGAGCTGTTCGGCACGCTGGGGGGCTCCGG
TATCCGTGCCCGCTAGTGCTTCTCTCAACCTGGCCCGCTTGTCGATTACCAACCTCCTCCT
GCAAGGACAGGGTGATTCTTGNATGCTTTACTATTCNTCCTCCTGGAAGGCTAAGGTGGG
TAAAGAGTCCACTATCNCTTGTGAGAACGGACAGACGGCGCAGTTATCCGATGANCATTC
AAGTCGTTGCCGAAAGTGTCTACATTGTCATCNAANAANAATATCGCTTCTTCCTTGCCT
GGCCCTAGTGTGAGTAGCGCCAATTCCTTCACCGACATATGAAAGGCGGCTGGCTTGAGT
TTCGAGGGTGTGCAACACGTCTCTTGAGGCACGAGTGATTATCTATTCTCANTCTGTTGT
GGGCTTTGATAAACAGACCCTACACGATGGTATAGTCTCTTACAAGGAATTTGGGATTATT
CACACTATGGACCTGCATGGGCGACGCGANTNCCTTCTGNCTATG-
ACANCTAACTTATTCCCTGACTTGNTTTCTATTGAGGNNNNNNATATCTGNNCCCG-
CTGGACGCTCTTCCA-
TGTGACGNGNGATCTATNACCCACCTTCTACTTCCTGTAGNANGAAGNCTATCNNATATC
ACGAATTACANTGNTCGTGNNANAGTTGCTGCGACTTCGACTNTGGTGCCCTGCCCAGA
GANGNNCCCGCAGCCNCNGACTACNAAACTCGATTTGGTCTCTAACATCTCACGTACAG
CTATATTCCTGAGGATNTNATCATCTACCAATGNNTCACCTTATCNNCT
>No18 Gastric
AAGTNTCTATATCAGCGAAGTGATGAGCCAACAAACCCAAATCAACACGGTAATTGAGC
GTTTTTATTCCCCTTTTTTAGAAGCTTTCCCCACTTTAAAAGATTTAGCGAACGCTCAATTA

GAAGAGGTTTTATTGCTCTGGAGAGGGCTTGGCTACTATTCAAGGGCTAAAAATTTAAAA
AAAAGCGCTGAAATTTGCGTTAAAGAGCATGACTCACAATTACCCAATGACTATCAAAGC
CTGTTGAAACTCCCCGGGATTGGCGCATACACGGCTAATGCGATTTTATGTTTTGGCTTTA
GAGAAAAGAGAGCATGCGTAGATGCTAACATCAAACGGGTGCTTTTAAGGCTTTTTGGTT
TGGATCCTAATATCACGGCTAAAGACTTACAAATTAAAGCGAATGACTTTCTCAATCTTAA
TGAAAGCTTTAATCATAACCAAGCCCTAATTGATCTAGGGGCTTTAATCTGCTCCCCTAAA
CCCAAATGCGCACTTTGCCCTTTCAATCCTTATTGTTTTAGGTAA
TAGGTTATTTNNNNNNNTGCGTTTGTTGTAAAAATTTTGTTTTGGAAGGATAAGGCAATG
CTAGGACTTGTATTGTTATATGTTGGGATTGTTTTAATCAGCAACGGGATTTGCGGATTAAC
CAAAGTCGATCCTAAAAGCACTGCGGTGATGAACTTTTTTGTGGGTGGGCTCTCCATTAT
TTGTAATGTGGTTGTCATCACTTATTCTGCGCTCAACCCTACAGCCCCTGTAGAAGGCGCA
GAGGATATTGCTCAAGTATCGCACCATTTGACTAATTTCTATGGGCCAGCAACTGGGTTAT
TGTTCGGTTTTACCTACTTGTATGCGGCTATCAACCACACTTTTGGTTTGGATTGGAGGCC
CTACTCTTGGTATAGCTTATTCGTAGCGATCAACACTGTTCCTGCTGCGATTTTATCCCACT
ATAGCGATATGCTTGATGACCACAAAGTGTTAGGCATCACTGAAGGCGATTGGTGGGCGA
TCATTTGGTTGGCTTGGGGTGTTTTGTGGCTTACCGCTTTCATTGAAAATATCTTGAAAAT
CCCTTTAGGGAAATTCACTCCATGGCTTGCTATCATTGAGGGTATTTTAACCGCTTGGATC
CCTGCTTGGTTACTCTTTATCCAACACTGGGTGTGAGATGATCATAGAGCGTTTAATAGGC
AATCTANGG
>No19 Gastric
TTNTTTTATATCAGCGAAGTGATGAGCCAACAAACCCAAATCAACACGGTAGTTGAGCGT
TTTTATTCCCCTTTTTTAAAAGCTTTCCCCACTTTAAAAGATTTAGCGAACGCTCCATTAG
AGGAGGTTTTATTGCTCTGGAGAGGGCTTGGCTATTATTCAAGGGCTAAAAATTTAAAAA
AAAGCGCTGAAATTTGCGTTAAAGAACACAACTCGCAATTACCCAATGACTATCAAAGCC
TGTTGAAACTCCCTGGGATTGGCGCATACACGGCTAATGCGATTTTATGTTTTGGCTTTAG
AGAAAAGAGCGCATGCGTGGATGCTAACATCAAACGAGTGCTTTTAAGGCTTTTTGGTTT
GGATCCAAATATCACGGCTAAAGACTTACAAATTAAGGCGAATGATTTTCTCAATCTCAAT
GAAAGCTTTAATCATAACCAAGCCCTAAATTGATCTAGGGGCTTTAATCTGCTCCCCTAAA
CCCAAATGCGTAATTTGCCCTTTCAATCCTTATTGGTTTAGGTAA
TTNGGTTATTTNNNNNNNTGCGTTTGTTGTAAAAATTTTTGTTTGGAAGGAAAAGGCAAT
GCTAGGACTTGTATTGTTATATGTTGGGATTGTTTTAATCAGCAATGGGATTTGCGGGTTAA
CCAAAGTCGATCCTAAAAGCACTGCGGTGATGAACTTTTTTGTGGGTGGGCTCTCCATTA
TTTGTAATATAGTTGTCATCACTTATTCTGCACTCCACCCTACAGCCCCTGTAGAAGGTGC
CGAAGATATTGCTCAAGTATCGCACCATTTGACTAGTTTCTATGGGCCAGCGACTGGGTTA
TTGTTCGGTTTTACCTACTTGTATGCGGCTATCAATCACACTTTTGGTTTGGATTGGAGGC
CCTACTCTTGGTATAGCTTATTCGTAGCGATCAACACTGTTCCTGCTGCGATTTTATCCCAC

TATAGCGATATGCTTGATGATCACAAAGTGTTAGGCATCACTGAAGGCGATTGGTGGGCG
ATCATTTGGTTGGCTTGGGGTGTTTTATGGCTTACCGCTTTCATTGAAAACATCTTGAAAA
TCCCTTTAGGGAAATTCACTCCATGGCTTGCTATCATTGAGGGTATTTTAACCGCTTGGAT
CCCTGCTTGGTTACTCTTTATCCAACACTGGGTGTGAGATGATCATAGAGCGTTTAATAGG
CAATCTAAGGNNAATTT

TACAACCGNNATCATTCCAGCCATTGTTGGGGGTATCGCTACAGGTGCTGCTGTAGGAAC
GGTCTCAGGGCTTCTTGGTTGGGGGCTCAAACAAGCCGAAGAAGCTAATAAAACCCCGG
ATAAACCCGATAAAGTTTGGCGCATTCAAGCAGGAAGAGGCTTTAATGAATTCCCTAACA
AGGAATACGACTTATACAAATCCCTTTTATCCAGTAAGATTGATGGAGGTTGGGATTGGGG
GAATGCCGCTAGGCATTATTGGGTCAAAGGTGGGCAATGGAACAAGCTTGAAGTGGATAT
GAAAGACGCTGTAGGGACTTATAAACTTTCAGGGCTAAGAAACTTTACCGGTGGGGATTT
AGATGTCAATATGCAAAAAGCCACTTTGCGTTTGGGCCAATTCAATGGCAATTCTTTCAC
AAGCTTTAAGGATAGCGCTGATCGCACCACGAGAGTGGATTTCAACGCTAAAAATATCTC
AATTGATAATTTTTTAGAAATCAATAACCGTGTGGGTTCTGGAGCCGGGAGAAAAGCCAG
CTCTACGGTTTTAACTTTGCAAGCTTCAGAGGGGATCACTAGTACAACCGNNATCATTCC
AGCCATTGTTGGGGGTATCGCTACAGGTGCTGCTGTAGGAACGGTCTCAGGGCTTCTTGG
TTGGGGGCTCAAACAAGCCGAAGAAGCTAATAAAACCCCGGATAAACCCGATAAAGTTT
GGCGCATTCAAGCAGGAAGAGGCTTTAATGAATTCCCTAACAAGGAATACGACTTATACA
AATCCCTTTTATCCAGTAAGATTGATGGAGGTTGGGATTGGGGGAATGCCGCTAGGCATTA
TTGGGTCAAAGGTGGGCAATGGAACAAGCTTGAAGTGGATATGAAAGACGCTGTAGGGA
CTTATAAACTTTCAGGGCTAAGAAACTTTACCGGTGGGGATTTAGATGTCAATATGCAAA
AAGCCACTTTGCGTTTGGGCCAATTCAATGGCAATTCTTTCACAAGCTTTAAGGATAGCG
CTGATCGCACCACGAGAGTGGATTTCAACGCTAAAAATATCTCAATTGATAATTTTTTAGA
AATCAATAACCGTGTGGGTTCTGGAGCCGGGAGAAAAGCCAGCTCTACGGTTTTAACTTT
GCAAGCTTCAGAGGGGATCACTAG

TCTGCTGTAGGGAACGGTCTCAGGGCTTCTTGGTTGGGGGCTCAAACAAGCCGAAGAAG
CGAATAAAACTCCGGATAAACCCGATAAAGTTTGGCGCATTCAAGCAGGAAGAGGTTTT
AATGAATTCCCTAACAAGGAATACGACTTATACAAATCCCTTTTATCCAGTAAGATTGATG
GAGGCTGGGATTGGGGGAATGCCGCTAGGCATTATTGGGTCAAAGGCGGGCAATGGAAC
AAGCTTGAAGTGGATATGAAAGACGCTGTAGGGACTTATAAGCTTTCAGGGCTAAGAAA
CTACACTGGTGGGGATTTAGATGTCAATATGCAAAAAGCCACTTTGCGTTTGGGCCAATT
CAATGGCAATTCTTTCACAAGCTTTAAGGATAGCGCTGATCGCACCACGAGAGTGGATTT
CAACGCTAAAAATATCTCAATTGATAATTTTTTAGAAATCAATAACCCGCGTGGGTTCTGG
AGCCGGCGAGAAAAGCCAGCTCCACGGTTTTAACTTTGCAAGCTTCAGAAGGGATCACT
AGCGGTAAAAACGCTGAAATTTCTCTTTATNNGNNNCCACGCA

>No19 Oral

TTATATCAGTGAAGTGATGAGCCAACAAACCCAAATCAACACGGTAATTGAGCGTTTTTA
TTCCCCTTTTTTAGAAGCTTTCCCCACTTTAAAAGATTTAGCGAACGCTCAATTAGAAGA
GGTTTTATTGCTCTGGAGAGGGCTTGGCTACTATTCAAGGGCTAAAAATTTAAAAAAAAG
CGCTGAAATTTGCGTTAAAGAGCATGACTCACAATTACCCAATGACTATCAAAGCCTGTT
GAAACTCCCCGGGATTGGCGCATACACGGCTAATGCGATTTTATGTTTTGGCTTTAGAGA
AAAGAGAGCATGCGTAGATGCTAACATCAAACGGGTGCTTTTAAGGCTTTTTGGTTTGGA
TCCTAATATCACGGCTAAAGACTTACAAATTAAAGCGAATGACTTTCTCAATCTTAATGAA
AGCTTTAATCATAACCAAGCCCTAATTGATCTAGGGGCTTTAATCTGCTCCCCTAAACCCA
AATGCGCACTTTGCCCTTTCAATCCTTATTGTTTAGGTAA
GCNCNCGGAGCANNNTGATTGTATTTTTTGTTGAATGTTNTAACCNCNGCGGTATGTGAG
CGTTGCGGACGATAGTCCTCGCCCGCGTGNTGTGAGTGAGAGTATGAGCATTGCCGTGC
AGCG--
TGGCTTGNTGATATGATTGAAATACTTCGGAGGCACATTGNCGTCCGTGGCACGGGGCTG
--CGGCTCCTTCCTCAAGAGCTCACGTATCCGATAGAGGATGTGCTTGCGCCCNANTCT---
CGGANGNNTGAAGAANTCGACTTGTTGGGTGAAGAA-
TATAGACCCGACCTTGTCTTTATGCTGAAACCCGGACAACGCTGGGCTTCTTGCATCTCN
AATTTGACTCGGGCTTGGCGATCTTCTTCACCACACCAGTCCGAACTCCCCGTCCCTGCG
AGAAGGANCATCAAGGTCATC--
TCCTATTCCACCTCTGAGACCTTGGGNTTNGAGNGGGCATAGCCGTGCTACGAATTCCAT
GAAATGCCTCTGACACGTCATCTCCTATCTGATAACCACCGCTGTCGGCCTTNNTCTTCTA
GCCTTGCCGACTTCTATCNNG-GGGACTGAGGATGNCCN-
GCAGACACTNGCTCTCAGAGNNCNCCGCCNCTTNNNNTCTGNGTNTCCNNNCNTNNNN
AAATACTCTCNN
>No20 Oral
CTCTTTGTTTTCAAACCCCTTGATATTAGCCATGACGCTGATCCTTTCCGCAAAATCTTCA
GCAGTCAGAGTGTAGGTCTTGCTGTTCGCTCCCCCAATTTTTTTGCCGTCGGGCAGCCAT
TGGTAGCTGACTTTGCTTTCGTCAAAGTTATCAGCGTCATGGATTTCGGCGGTCAGGGTC
TCCCCGACCTTGGCGTTACCAGTGATAGTTACGGTGCCTTCGTGATTAGGGTCAGGCGGG
GTTTGCCCGTCAGTGACAGCTTCAGTTTCCCCACTGGGCGGGGTTTCATTGTGCTGTGCA
TTATCGGTGTAAGTTGCTTTGACACTGATTTTCTGGCCAACATCATTGACGGTCAGGGGG
TAGGCCTTGCCGTTAGCGCCGGCGATTTCTTTGCCCTCGGGCAGCCATTGGTAGCGGACT
TTTTTTTCAAAGAATAAG
ACAAGTAAAATGAATACAAGCCATAAAACTTTAAAAACCATTGCGATTTTAGGCCAGCCT
AATGTGGGGAAAAGCTCGTTATTTAACCGCCTGGCTAGAGAAAGGATCGCTATCACTTCA
GATTTTGCAGGCACTACACGAGACATTAACAAACGAAAAATCGCATTGAATGGCCATGA
AGTGGAATTGCTAGATACAGGGGGCATGGCTAAAGACGCTCTTTTGTCTAAAGAAATCAA

AGCCCTTAATTTAAAAGCCGCTCAAATGAGCGATTTGATTTTATATGTTGTGGATGGTAAG
TCTATCCCTAGCGATGAAGATCTTAAGCTTTTTAGAGAGGTTTTTAAGATCAACCCTAACT
GCTTTTTAGTGATCAATAAAATTGACAACGACAAAGAAAAAGAGCGAGCTTATGCATTTT
CTTCTTTTGGCATGCCAAAGAGTTTTAATATTTCTGTTTCGCACAATAGAGGCATTAGTGC
CCTAATTGATGCGATATTGAACGCGCTAAATTTAAATCAAATCATAGAGCAGGATTTGGAT
GCGGATATTTTAGAAAGCCTAGAAAATAACGCATCAAAAGAAGAACTT
>No20 Gastric
TTAGCCATGACGCTGAGTCTTTGTGCGTGGTGATTGAAATATCCAAGCATTCTAATATCAA
GTATGAACTGGATAAAGAAAGCGGGGCTTTAATGGTGGATAGGGTGCTTTATGGGGCGCA
AAATTACCCCGCCAATTATGGCTTTGTGCCTAACACTCTAGGATCTGATGGCGACCCTGTA
GATGCGCTTGTTTTAAGCGATGTGGCTTTTCAAGCCGGGAGCGTGGTGAAAGCGCGCTT
GGTTGGGGTTTTGAACATGGAAGATGAAAGCGGAATGGATGAAAAATTACTTGCTCTGC
CCATAGATAAAATCGATCCCACGCATTCTTATGTCAAAGATATTGATGATTTATCCAAACAC
ACTTTAGATAAAATCAAGCATTTTTTTGAAACTTACAAGGATTTAGAGCCTAATAAATGGG
TGAAAGTCAAGGGGTTTGAAAAACAAAGAGA
CCATAAAACTTTAAAAACCATTGCGATTTTAGGCCAGCCTAATGTGGGGAAAAGCTCGTT
ATTTAACCGCTTGGCTAGAGAAAGAATCGCTATCACTTCAGATTTTGCAGGCACTACACG
AGACATTAACAAACGAAAAATCGCATTGAATGGCCATGAAGTGGAATTGCTAGATACAGG
GGGCATGGCTAAAGACGCTCTTTTGTCTAAAGAAATCAAAGCCCTTAATTTAAAAGCCGC
TCAAATGAGCGATTTGATTTTATATGTTGTGGATGGCAAGTCTATCCCTAGCGATGAAGAT
CTTAAGCTTTTTAGAGAAGTTTTTAAAACCAACCCTAACTGCTTTTTAGTGATCAATAAAA
TTGATAACGACAAAGAAAAAGAGCGAGCTTATGCGTTTTCTTCTTTTGGCATGCCAAAGA
GTTTTAATATTTCCGTTTCGCACAATAGAGGCATTAGTGCCTTAATTGATGCGATATTGAGC
ACGCTAAATTTAAATCAAATCATAGAGCAGGATTTGGATGCGGATATTTTAGAAAGCCTAG
AAAATAAACGCAT
>No21 Oral
GTTATTNNNTAAGGTGCGTTTGTTGTAAAAATTTTGTTTTGGAAGGATAAGGCAATGCTAG
GACTTGTATTGTTATATGTTGGGATTGTTTTGATCAGCAATGGGATTTGCGGATTAACCAA
AGTCGATCCTAAAAGCACTGCGGTGATGAACTTTTTTGTGGGTGGGCTTTCCATTATTTGT
AATGTGGTTGTCATCACTTATTCTGCACTCAACCCTACAGCCCCTGTAGAAGGTGCAGAA
GATATTGCTCAAGTATCGCACCATTTGACTAATTTCTATGGGCCAGCAACTGGGTTATTGTT
CGGTTTTACCTACTTGTATGCGGCTATCAACCACACTTTTGGTTTGGATTGGAGGCCCTAC
TCTTGGTATAGCTTATTCGTAGCGATCAACACTGTTCCTGCTGCGATTTTATCCCACTATAG
CGATATGCTTGATGACCACAAAGTGTTAGGCATCACTGAAGGCGATTGGTGGGCGATCAT
TTGGTTGGCTTGGGGTGTTTTGTGGCTTACCGCTTTCATTGAAAACATCTTAAAAATCCCT
TTAGGGAAATTCACTCCATGGCTTGCTATCATTGAGGGCATTTTAACCGCTTGGATCCCTG

CTTGGTTACTCTTTATCCAACACTGGGTGTGAGATGATCATAGAGCGTTTAATGGGCAATC
TAAGGAANTTTTAAANA
TGAAACTACAAGTAAAATGAATACAAGCCATAAAACTTTAAAAACCATTGCGATTTTAGG
CCAGCCTAATGTGGGGAAAAGCTCGTTATTTAACCGCTTGGCTAGAGAAAGGATCGCTAT
CACTTCAGATTTTGCAGGCACTACACGAGACATTAACAAACGAAAAATCGCATTGAATGG
CCATGAAGTGGAATTGCTAGATACAGGGGGCATGGCTAAAGACGCTCTTTTGTCTAAAGA
AATCAAAGCCCTTAATTTAAAAGCCGCTCAAATGAGCGATTTGATTTTATATGTTGTGGAT
GGCAAGTCTATCCCTAGCGATGAAGATCTTAAGCTTTTTAGAGAGGTTTTTAAAACCAAC
CCTAACTGCTTTTTAGTGATCAATAAAATTGATAACGACAAAGAAAAAGAGCGAGCTTAT
GCGTTTTCTTCTTTTGGCATGCCAAAGAGTTTTAATATTTCTGTTTCGCACAATAGAGGCA
TTAGCGCCTTAATTGATGCGATATTGAACGCGCTAAATTTAAATCAAATCATAGAGCAGGA
TTTGGATGCGGATATTTTAGAAAGCCTAGAAAATAACGCA
>No21 Gastric
TGCGTTTGTTGTAAAAATTTTGTTTTGGAAGGATAAGGCAATGCTAGGACTTGTATTGTTA
TATGTTGGGATTGTTTTAATCAGCAACGGGATTTGCGGATTAACCAAAGTCGATCCTAAAA
GCACTGCGGTGATGAACTTTTTTGTGGGTGGGCTTTCCATTGTTTGTAATGTGGTTGTCAT
CACTTATTCTGCGCTCAACCCTACAGCCCCTGTAGAAGGCGCAGAAGATATTGCTCAAGT
ATCGCACCATTTGACTAATTTCTATGGGCCAGCAACTGGGTTATTGTTCGGTTTTACCTACT
TGTATGCGGCTATCAACCACACTTTTGGTTTGGACTGGAGGCCCTACTCTTGGTATAGCTT
ATTCGTAGCGATCAACACTGTTCCTGCTGCGATTTTATCCCACTATAGCGATATGCTTGATG
ACCACAAAGTGTTAGGCATCACTGAAGGCGATTGGTGGGCGATCATTTGGTTGGCTTGGG
GTGTTTTGTGGCTTACCGCTTTCATTGAAAATATCTTGAAAATCCCTTTAGGGAAATTCAC
TCCATGGCTTGCTATCATTGAGGGTATTTTAACCGCTTGGATTCCTGCTTGGTTACTCTTTA
TCCAACACTGGGTGTGAGATGATCATAGAGCGTTTAATGGGCAATCTAAGGGNAANTTAA
ACA
GAACTACAAGTAAAATGAATACAAGCCATAAAACTTTAAAAACCATTGCGATTTTAGGCC
AGCCTAATGTGGGGAAAAGCTCGTTATTTAACCGCTTGGCTAGAGAAAGGATCGCTATCA
CTTCAGATTTTGCAGGCACTACACGAGACATTAACAAACGAAAAATCGCATTGAATGGCC
ATGAAGTGGAATTGCTAGATACAGGGGGCATGGCTAAAGACGCTCTTTTGTCTAAAGAAA
TCAAAGCCCTTAATTTAAAAGCCGCTCAAATGAGCGATTTGATTTTATATGTTGTGGATGG
CAAGTCTATCCCTAGCGATGAAGATCTTAAACTTTTTAGAGAGGTTTTTAAAACCAACCCT
AACTGCTTTTTAGTGATCAATAAAATTGATAACGACAAAGAAAAAGAGCGAGCTTATGCG
TTTTCTTCTTTTGGCATGCCAAAGAGTTTTAATATTTCTGTTTCGCACAATAGAGGCATTAG
CGCCTTAATTGATGCGATATTGAACGCGCTAAATTTAAATCAAATCATAGAGCAGGATTTG
GATGCGGATATTTTAGAAAGCCTAGAAAATAA
